# Dynamic asymmetric spillovers and connectedness between Chinese sectoral commodities and industry stock markets

**DOI:** 10.1371/journal.pone.0296501

**Published:** 2024-01-02

**Authors:** Yu Lou, Chao Xiao, Yi Lian

**Affiliations:** 1 School of Finance, Southwestern University of Finance and Economics, Chengdu, Sichuan, China; 2 School of Economics and Management, Anyang University, Xinxiang, Henan, China; Bucharest University of Economic Studies: Academia de Studii Economice din Bucuresti, ROMANIA

## Abstract

This study investigates the dynamic and asymmetric propagation of return spillovers between sectoral commodities and industry stock markets in China. Using a daily dataset from February 2007 to July 2022, we employ a time-varying vector autoregressive (TVP-VAR) model to examine the asymmetric return spillovers and dynamic connectedness across sectors. The results reveal significant time-varying spillovers among these sectors, with the industry stocks acting as the primary transmitter of information to the commodity market. Materials, energy, and industrials stock sectors contribute significantly to these spillovers due to their close ties to commodity production and processing. The study also identifies significant asymmetric spillovers with bad returns dominating, influenced by major economic and political events such as the 2008 global financial crisis, the 2015 Chinese stock market crisis, the COVID-19 pandemic, and the Russia-Ukraine war. Furthermore, our study highlights the unique dynamics within the Chinese market, where net information spillovers from the stock market to commodities drive the financialization process, which differs from the bidirectional commodity financialization observed in other markets. Finally, portfolio analysis reveals that the minimum connectedness portfolio outperforms other approaches and effectively reflects asymmetries. Understanding these dynamics and sectoral heterogeneities has important implications for risk management, policy development, and trading practices.

## 1 Introduction

This century has seen an influx of financial investors into the commodity markets, significantly changing the composition of commodity assets in their portfolios. Commodities have evolved from serving their traditional role of hedging risk to support the real economy to becoming attractive investment vehicles for diversification and risk management [1]. This transformation has amplified investors’ influence on commodity markets and increased the connectedness between commodity markets and other capital markets. Commodity prices have shifted from being driven primarily by the supply and demand dynamics of economic fundamentals to being increasingly influenced by other financial capital markets [2–5]. Scholars have referred to this trend as the financialization of commodities.

This financialization process has significantly intensified the interconnectedness between commodity markets and capital markets, especially stock markets. Traditionally, commodity and stock markets were considered distinct domains, with commodity prices predominantly influenced by natural factors such as climate and geography, while stocks are valued based on discounted future cash flows. However, the rise of financialization has blurred these lines, fostering mechanisms like information frictions and spillovers that accelerate the integration of these markets, especially during crises (i.e., the 2008 global financial crisis, the 2015 Chinese stock market crisis, the COVID-19 pandemic, and the Russia-Ukraine war). Theories such as the fractal market hypothesis and market integration theory provide frameworks for understanding these evolving dynamics. The fractal market hypothesis posits financial markets as sophisticated stochastic processes with interactive and adaptive properties, acknowledging that market information and investment timeframes can alter investor behavior and thus drive market efficiency [6]. Market integration theory, on the other hand, suggests that prices in one market are influenced by those in other markets [7]. This intricate network of financial interconnectedness has drawn increased attention to the intersection of commodity and stock markets.

In addition, the presence of asymmetry in asset price movements is a well-established phenomenon directly related to market dynamics influenced by positive and negative innovations, which not only has important implications for market dynamics, but have also become a major concern for investors [1, 8–10]. Nevertheless, the recent empirical literature has paid limited attention to the intensity and direction of positive and negative return spillovers. This asymmetric connection warrants comprehensive exploration, especially in light of the ongoing financialization of commodities and the deepening interconnectedness between commodity and stock markets. In particular, negative shocks have the potential to amplify asset price volatility, depress valuations, and intensify cross-market risk contagion, thereby heightening overall market instability. Conversely, positive shocks stimulate investment activity, contribute to asset price increases, and strengthen price transmission across markets [11, 12]. Studying the asymmetric linkages between commodity and stock markets can provide valuable insights into the complex and dynamic relationship between these sectors. This knowledge is essential for investors and policymakers to identify potential risk transmission channels, effectively manage portfolio risk, and maintain market stability in the face of unexpected events.

Exploring asymmetric return spillovers and connectedness between commodity and stock markets holds profound significance and value, particularly for the Chinese market. First, China has emerged as a major consumer of global commodities. As of 2021, seven of the top ten commodity futures contracts by trading volume were traded on Chinese exchanges. With substantial commodity consumption demand, growing investor interest, and advancing financial liberalization, the Chinese commodity market plays a crucial role in shaping global commodity prices and dynamics [13, 14]. However, due to entry barriers, national influences, and unique trading rules, the Chinese commodity futures market remains a mystery to practitioners and scholars [15]. Thus, attracting more attention from investors and scholars to the Chinese commodity market is imperative. Second, the Chinese commodity market is characterized by a number of unique features, such as the prevalence of retail investors, the lack of commodity investment vehicles, and the complex interplay between state and market forces. These market conditions make it difficult for China to replicate the phenomenon of commodity financialization, as seen in developed markets. Nevertheless, according to the existing literature, a certain degree of commodity financialization is still evident in the Chinese commodity market, albeit to a lesser extent than in developed markets [16]. Moreover, it is important to note that the degree of financialization varies across different sectors within the commodity market. Given the nascent stage of commodity financialization in the Chinese market and the partial segmentation that still exists among different sectoral commodities, the price fluctuations of individual commodities fail to significantly affect the entire stock market or even specific stock sectors. At present, many scholars have based their research on the spillover effects between individual commodity futures and stock markets, which is not applicable to the Chinese market. Therefore, a more detailed examination of the connectedness between the commodity and stock markets at the sector level is necessary to understand the unique path of commodity financialization in China and the sources of differences in financialization across different sectors [16]. This will help investors who prefer diversified investment portfolios to identify cross-market sector reactions. Finally, the existing body of research on the relationship between the commodity market and the stock market in China remains limited, particularly at the sector level. While extensive studies have examined the relationship between commodity and stock markets in mature economies, the unique characteristics of the Chinese market have not received commensurate attention. This research gap is further exacerbated when considering the presence of asymmetric return spillovers between these markets. Therefore, filling this knowledge gap is crucial not only for market stability, risk assessment, and the formulation of effective investment strategies but also for providing valuable insights into the causes of commodity financialization in China.

Given these factors, this paper examines the dynamic asymmetric return spillovers and connectedness between the sector-level agricultural futures index, metals futures index, chemical products futures index, energy futures index, and 11 sector stock indices (energy, materials, industrials, major consumer goods, discretionary consumer goods, healthcare, finance, real estate, information technology, communication services, and utilities sectors) in the Chinese market. The aim is to fill the gap in research on the asymmetric relationship between the Chinese commodity market and the stock market while providing sector-level connectedness as a way to explain the formation of commodity financialization in China. In the empirical study, we employ the time-varying parameter vector autoregressive (TVP-VAR) model recently introduced by Antonakakis et al. [17] and the pairwise connectedness index methodology proposed by Gabauer [18] to examine the association between Chinese commodity and stock markets at the sectoral level within the network topology framework established by Diebold and Yilmaz [19–21]. In this framework, we can estimate the static and dynamic spillover effects across sectoral markets and measure the strength and direction of time-varying return spillover contributions between commodity and stock market sectors. In addition, we decompose the total returns of each sector into positive and negative returns based on the different reactions of investors to market upturns and downturns. This allows us to analyze the positive and negative return spillovers between commodity and stock sectors and to assess the asymmetric spillover characteristics between Chinese sectoral commodities and industrial stocks. Our empirical results show significant and dynamic asymmetry return spillovers between China’s different sectoral commodities and industry stocks. Specifically, commodities exhibit net recipient behavior with sector-dependent variations, and the Materials (MT), Energy (EG), and Industrials (IND) sectors were found to have the most significant impact on return spillovers to commodity markets. Moreover, major political and economic events significantly affected these asymmetric return spillovers, leading to changes in the intensity and direction of spillovers across sectors.

Therefore, the contribution of this paper is fourfold. First, our study extends the existing literature on price linkages between commodity and stock market sectors. Unlike previous studies that focus on the interactions between a specific commodity in a category and the stock market [17, 22–26], this study analyzes the interconnectedness of markets from a sectoral commodity perspective. This study reduces the impact of idiosyncratic factors affecting specific or individual commodities on a basket of different commodity sector indices and uncovers sector heterogeneity that may be obscured when analyzing aggregate stock indices. Second, based on the stylized fact of asymmetric behavior of asset price movements, we distinguish between negative and positive return spillovers across commodity and stock market sectors and identify the evolution of cross-sector asymmetric spillovers under the influence of major economic and political events (i.e., the 2008 global financial crisis, the 2015 Chinese stock market crisis, the COVID-19 pandemic, and Russia-Ukraine war). In contrast to the current literature on asset price linkages between China’s commodity and stock markets, which rarely focuses on asymmetric spillovers [27–31], we extend the existing literature by introducing the concept of asymmetric return spillovers into the analysis of the dependence between China’s commodity and stock markets. This also provides an opportunity to estimate portfolio and diversification returns based on asymmetric spillovers (including negative and positive spillovers). Third, this study favors the sectoral commodity perspective over previous studies on the interdependence of commodity and stock markets in China [27, 28]. By dissecting the connectedness between commodity and stock markets at the sectoral level, this study provides insights into the information transmission pathways in the evolving interconnections between commodity and stock markets during the nascent stage of China’s commodity financialization, which effectively complements the literature on the financialization of China’s commodity market. Finally, in terms of empirical methods, we use the TVP-VAR approach, which has advantages over the rolling window estimation of Diebold and Yilmaz [19–21] in previous studies. It does not require a choice of rolling window size, is less sensitive to extreme outliers, and therefore does not result in loss of observations to better adapt to parameter variations. It is also more accurate in calculating the generalized forecast error variance decomposition (GFEVD).

The remainder of this study is structured as follows. Section 2 provides a brief overview of the literature. Section 3 presents our empirical methodology. Section 4 describes the data and provides some preliminary analysis of the data. Section 5 provides the empirical results and discussion. Finally, section 6 concludes the paper.

## 2 Brief literature review

The complex relationship between commodities and the stock market has been the focus of considerable research, mainly because of its important implications for risk management, portfolio diversification, and financial stability. Early studies predominantly focused on the relationship between individual commodities and the stock market, with oil–a strategic commodity closely tied to economic development and monetary policy–receiving particular attention due to the earliest observed spillover effects on stock market returns [25, 32].

With the development and liberalization of financial markets, the speed of information transmission across different markets and even different asset categories has increased significantly. This phenomenon led Tang and Xiong [5] to introduce the concept of commodity financialization in their influential paper. As commodity financialization expands, academic interest has increased in the interconnections between commodity and stock markets. This extends to a broader spectrum of individual commodity types, commodity sectors, and their interactions with the stock market and its various sectors.

In this literature, the first strand of research employs multivariate GARCH models, VAR or ARCH-type models, and tail-dependency measures to examine the dynamic conditional correlation, the transmission of returns or volatilities, and the dependence structure (upper and lower tail dependence) between commodity and stock markets [23, 33–46]. Nevertheless, these econometric methods face challenges in quantifying spillover relationships in detail [36]. Diebold and Yilmaz [19–21] introduced a method to quantitatively measure spillover effects through connectedness based on the variance decomposition of vector autoregression forecast errors. Another strand of the literature utilizes the Diebold and Yilmaz [19–21] approach and its various extensions to study bilateral and multilateral spillovers between different assets in the commodity and stock markets [10, 17, 24, 25, 32, 47–54]. Additionally, with the decline in diversified returns in the stock market, equity investors have become more proactive in seeking hedging positions in commodity assets during turbulent periods. In the increasingly interconnected relationship between commodity and stock markets, asymmetric spillovers have received particular attention in recent research. The quantitative measurement of time-varying asymmetric spillover effects based on the method of Diebold and Yilmaz [19–21] has also begun to receive academic attention. Papers by Palanska [11], Xu et al. [32], Suleman et al. [24], and Adekoya et al. [17], applying the Diebold and Yilmaz’s [19–21] method and its extensions, have documented the asymmetric features of spillovers between individual commodities and stock markets, as well as sectoral stocks, in response to positive and negative shocks. Consistently, they find that aside from the early stages of the COVID pandemic, pre-crisis asset spillovers were limited, but negative shocks typically dominated after the crisis. Gabauer et al. [55] argue that in addition to financial crises, health crises can also have a negative impact on commodity returns, a phenomenon that our results confirm. The onset of the COVID-19 pandemic in 2020 has increased the risk and uncertainty in commodity and stock markets. Ashraf et al. [56], Amar et al. [57], Farid et al. [58], Mezghani et al. [59], and Liu et al. [26] also documented the negative impact and uncertainty shocks of the pandemic on commodity and stock markets, which intensified the spillovers across markets. In addition, Apergis et al. [60] showed that news sentiment related to the COVID-19 pandemic dominated the spillover shocks between commodity and stock markets, serving as the most potent net transmitter of uncertainty.

Although the literature has long recognized the presence of asymmetric spillover effects between commodity and stock markets in developed financial markets, research investigating similar dynamics within the increasingly important Chinese market in the global commodity landscape remains limited. Kang and Yoon [29] explored the dynamics of return and volatility spillovers across Chinese stocks and four commodity futures, employing the multivariate DECO-GARCH and the spillover index models. Their research provided empirical evidence of enhanced return and volatility spillovers during financial crises. In parallel, focusing on the relationship between individual commodities and the stock market, Ding et al. [31] used a VAR model to examine the impact persistence of volatility spillover and illiquidity spillover within Chinese commodity markets. Their findings underscore that different commodity markets exhibit different responsiveness to stock market shocks, reflecting their unique market characteristics. Furthermore, from a sectoral perspective, Wen et al. [30] investigated the relationship between nine commodity sectors and the stock market. They found a significant interrelation between the Chinese stock and commodity markets. On the other hand, Mensi et al. [25] conducted a study on the time-varying asymmetric spillovers between gold futures, West Texas Intermediate (WTI) crude oil futures, and ten different Chinese stock sectors. Their findings revealed evidence of negative return spillovers from international gold and oil dominating the spillovers.

From the foregoing, it is apparent that in addition to the insufficient focus on the Chinese commodity market, the current research on the study of dynamic spillovers between Chinese commodity and stock markets is limited in several aspects: (a) the consideration of asymmetry in the spillovers analysis, (b) the sectoral heterogeneity in spillover analysis between commodity and stock markets has not been sufficiently emphasized, and the scope of research sample is not comprehensive, (c) the explanation for the nascent stage of commodity financialization in the Chinese market based on the information transmission channels between commodity and stock markets at the sectoral level. Our research attempts to fill these gaps by investigating the dynamic aggregate connectedness and the asymmetric spillovers between sectoral commodities and industry stocks in China. We include all sectors in China’s commodity and stock markets in our analysis and take into account the impact of major economic and political events, aiming to provide a nuanced understanding of the interactions between these markets. This effort contributes to a broader understanding of market dynamics and can potentially inform policy decisions and strategic investment planning.

## 3 Methodology

### 3.1 TVP-VAR based asymmetric connectedness approach

Diebold and Yilmaz [19–21] (hereafter, DY) developed the connectedness approach that utilized the variance decomposition of the forecast errors in a vector autoregressive model (VAR) to measure the magnitude of the connectedness effect in the time domain. That is, measure connectedness by recording how much of the ij-th H-step variance decomposition component of $d_{ij}^{H}$, i.e., the fraction of the variable i’s H-step forecast error variance due to innovations in another variable j. This method reveals complex nonlinear transport mechanisms in multi-subject connected networks in the connectedness table through the VAR models, namely IRFs and FEVDs.

Much research has been built on Diebold and Yilmaz’s [19–21] innovative and widely applicable method, but it still has many limitations for different scenarios. Among them, Antonakakis et al. [61] improved Diebold and Yilmaz’s [19–21] method by using the TVP-VAR method instead of the rolling window VAR, which solved the problems of depending on a rolling window size and being insensitive to outliers in the prediction error variance decomposition.

To examine the asymmetric return spillovers and connectedness between sectoral commodities and industry stocks in China, we attempt to explore time-varying returns transmission mechanisms between the commodity market and the stock market. Based on Antonakakis et al. [61], we employ the TVP-VAR for positive and negative index returns to measure the asymmetry of connectedness between sectoral commodities and industry stocks. The TVP-VAR model with first-order lag length suggested by the Bayesian information criterion (BIC) can be written as follows,

$$y_{t}=A_{t}y_{t-1}+\varepsilon_{t}\;\varepsilon_{t}∼N(0,\Sigma_{t}),$$ (1)

$$vec(A_{t})=vec(A_{t-1})+u_{t}\;u_{t}∼N(0,\Xi_{t}).$$ (2)

Where *y*_*t*_, *ε*_*t*_ and *u*_*t*_are *K*×1 dimensional vector and *A*_*t*_ and Σ_*t*_ are *K*×*K* dimensional matrics for the time-varing VAR coefficients and variance-covariances. *vec*(*A*_*t*_) and *u*_*t*_ are *K*^2^×1 dimensional vector as well as Ξ_*t*_ is a *K*^2^×*K*^2^ dimensional matrix. The relationships across series are allowed to vary over time, also including Σ_*t*_ and Ξ_*t*_. Then, the time-varying coefficients and variance-covariance matrics are estimated using the generalized impulse response functions (GIRF) and generalized forecast error variance decompositions (GFEVD) proposed by Koop et al. [62] and Pesaran and Shin [63], respectively, based on the process of Diebold and Yilmaz [20] for connectedness estimation. To calculate the GIRF and GFEVD, we transform the TVP-VAR model based on Wold’s theorem into the following equation expression for the TVP-VMA: $y_{t}=\sum_{h=0}^{\infty }B_{h,t}\varepsilon_{t-i}$ where *B*_0_ = *I*_*K*_.

We focus on GFEVD. Diebold and Yilmaz [19–21] scale GFEVD by dividing the unscaled GFEVD by the row sum so that each row adds up to unity. Hence, GFEVD (${\tilde{\phi }}_{ij,t}^{g}(h)$) represents pairwise directional connectedness from j to i, meaning the influence variable j exerts on variable i based on its forecast error variance share. We calculate this by:

$$\phi_{ij,t}^{g}(h)=\frac{\Sigma_{ii,t}^{-1}\sum_{t=1}^{h-1}{\left(I_{i}^{\prime }A_{t}\Sigma_{t}I_{j}\right)}^{2}}{\sum_{j=1}^{k}\sum_{t=1}^{h-1}\left(I_{i}A_{t}\Sigma_{t}A_{t}^{\prime }I_{i}\right)},$$ (3)

$${\tilde{\phi }}_{ij,t}^{g}(h)=\frac{\phi_{ij,t}^{g}(h)}{\sum_{j=1}^{k}\phi_{ij,t}^{g}(h)}.$$ (4)

With $\sum_{j=1}^{k}{\tilde{\phi }}^{g}{}_{ij,t}(h)=1$ and $\sum_{i,j=1}^{k}{\tilde{\phi }}^{g}{}_{ij,t}(h)=k$, where ${\tilde{\phi }}_{ij,t}^{g}(h)$ stand for h-step ahead GFEVD, **Σ**_*t*_ Σ_t_ is the variance matrix of the error vector *ε*_*t*_ and ***I***_*i*_ is the selection vector, with unity on the ith postion and zeros otherwise.

Based on the scaled GFEVD, we calculate the total connectedness index as a measure of the interconnectedness of networks with the following formula:

$$C_{t}^{g}(h)=\frac{\sum_{i,j=1,i\neq j}^{k}{\tilde{\phi }}_{ij,t}^{g}(h)}{\sum_{i,j=1}^{k}{\tilde{\phi }}_{ij,t}^{g}(h)}.$$ (5)

This total connectedness index measures the average contribution of spillovers of shocks across all variables to the total forecast error variance. It reflects the average spillover proportion of shocks to one variable over the other.

We also focus on a scenario where variable i is subject to a shock from all other variables j, expressed as total directional connectedness from others (abbreviated as FROM directional connectedness), with the following formula:

$$C_{i\leftarrow j,t}^{g}(h)=\sum_{j=1,i\neq j}^{k}{\tilde{\phi }}_{ij,t}^{g}(h).$$ (6)

In turn, variable i, which transmits its shocks to all others, is expressed as total directional connectedness to others (abbreviated as To directional connectedness) by the following formula:

$$C_{i\to j,t}^{g}(h)=\sum_{j=1,i\neq j}^{k}{\tilde{\phi }}_{ji,t}^{g}(h).$$ (7)

Net directional connectedness can therefore be defined by subtracting the total directional connectedness to others from the total directional connectedness from others, which represents the extent to which a variable is affected by or affects other variables:

$$C_{i,t}^{g}(h)=C_{i\to j,t}^{g}(h)-C_{i\leftarrow j,t}^{g}(h).$$ (8)

Specifically, if net directional connectedness is negative, it indicates that variable i is a net recipient of shocks from other variables in that network; otherwise, it is a net transmitter of other variables in the network.

In addition, We need to measure the bidirectional connectedness further to provide information on whether variable i is a transmitter or a recipient of variable j at a more disaggregated level. Therefore, the concept of net pairwise directional connectedness (NPDC) is defined by:

$$NPDC_{ij}(h)={\tilde{\phi }}_{ji,t}^{g}(h)-{\tilde{\phi }}_{ij,t}^{g}(h).$$ (9)

Finally, reconsidering the problem of the existence of TCI, Chatziantoniou and Gabauer [64] found TCI within $\left[0,\frac{k-1}{k}\right]$, and interpretation of the high interconnectedness to be subjective, and adjusted the TCI accordingly:

$$\begin{array}{l} C_{t}^{g}(H)=(\frac{k}{k-1})\frac{\sum_{i,j=1,i\neq j}^{k}{\tilde{\phi }}_{ij,t}^{g}(h)}{k}\\ \;=\frac{\sum_{i,j=1,i\neq j}^{k}{\tilde{\phi }}_{ij,t}^{g}(h)}{k-1}\;0\leq C_{t}^{g}(h)\leq 1. \end{array}$$ (10)

The pairwise connectedness index (PCI) measures the interconnectedness across two variables *i* and *j*, as shown by Gabauer [18]:

$$\begin{array}{l} C_{ijt}^{g}(H)=2(\frac{{\tilde{\phi }}_{ij,t}^{g}(h)+{\tilde{\phi }}_{ji,t}^{g}(h)}{{\tilde{\phi }}_{ii,t}^{g}(h)+{\tilde{\phi }}_{ij,t}^{g}(h)+{\tilde{\phi }}_{ji,t}^{g}(h)+{\tilde{\phi }}_{jj,t}^{g}(h)})\\ \;0\leq C_{ijt}^{g}(h)\leq 1. \end{array}$$ (11)

### 3.2 Multivariate portfolio construction

To assess the financial implications of our empirical findings, we employ various multivariate portfolio construction techniques, assuming that investors have access to investable trackers or equivalent investment vehicles.

We begin with the widely accepted the minimum variance portfolio (MVP) method [65], which aims to construct a portfolio with the lowest volatility given multiple financial assets. The portfolio weights are determined using the following equation:

$$w_{\Sigma_{t}}=\frac{\Sigma_{t}^{-1}I}{I\Sigma_{t}^{-1}I}$$ (12)

where $w_{\Sigma_{t}}$ represents the *m*×1 portfolio weight vector, *I* is an m-dimensional vector of ones, and Σ_t_ represents the *m*×*m* conditional variance-covariance matrix in period *t* from the TVP-VAR model.

Next, we employ the minimum correlation portfolio (MCP) method [66], a recently developed approach. Unlike the MVP, MCP uses conditional correlation matrices instead of covariance matrices to determine portfolio weights. The optimal portfolio weights are calculated as follows:

$$R_{t}=diag{\left(\Sigma_{t}\right)}^{‐0.5}\Sigma_{t}diag{\left(\Sigma_{t}\right)}^{‐0.5}$$ (13)

$$w_{R_{t}}=\frac{R_{t}^{-1}I}{IR_{t}^{-1}I}$$ (14)

where *R*_*t*_ is an *m*×*m* conditional correlation matrix.

Finally, we construct the minimum connectedness portfolio (MCoP) using the pairwise connectedness indices [67]. This approach minimizes the connectedness between assets by assigning higher weights to assets that have less influence on, and are less susceptible to, other assets. This results in a more resilient portfolio, less susceptible to network shocks. The formulation is as follows:

$$w_{C_{t}}=\frac{PCI_{t}^{‐1}I}{IPCI_{t}^{‐1}I}$$ (15)

where *PCI*_*t*_ is the pairwise connectedness index matrix, and *I* is the identity matrix.

Additionally, we evaluate portfolio performance by two key metrics: the sharp ratio [68] and hedge effectiveness [69]. The Sharpe ratio measures risk-adjusted returns and is computed as:

$$SR=\frac{{\bar{r}}_{p}}{\sqrt{\mathrm{var}(r_{p})}}$$ (16)

where ${\bar{r}}_{p}$ is the portfolio return. The higher the SR, the higher portfolio return ${\bar{r}}_{p}$ for portfolio risk. Hedge effectiveness assesses the degree of risk reduction in a portfolio relative to a single asset. It is calculated as:

$$HE=1-\frac{Var_{p}}{Var_{i}}$$ (17)

where *Var*_*p*_ is the portfolio variance and *Var*_*i*_ is the variance of the asset *i*. Higher HE ratios mean greater risk reduction, indicating a more effective hedge against market volatility.

## 4 Data and preliminary analysis

### 4.1 Data

To analyze the asymmetric return spillovers and connectedness between Chinese commodities and sector stocks, we employ a daily dataset from the WIND database that includes the CSI Commodity Sector Composite Index and the CSI 800 Industry Index (CSI 800) from January 2007 to August 2022 with 3808 daily observations. These sectors encompass a wide range of industries, including the Energy (EG), Materials (MT), Industrials (IND), Consumer Discretionary (CD), Consumer Staples (CSP), Healthcare (HTH), Financials (FIN), Real Estate (RE), Information Technology (IT), Communication Services (CS), and Utility (UTI) sector. The CSI 800 Index provides comprehensive coverage of the Chinese stock market by including 800 large, medium, and small companies listed in the CSI 300 and CSI 500 indices. On the other hand, the CSI Commodity Sector Composite Index categorizes commodity futures into four distinct commodity sectors: Agriculture (AG), Metals (MET), Chemical Products (CMP), and Energy (ERG) futures sectors. These futures sectors covered 63 commodity futures listed in China and represent the key components of the commodity market in China. In this study, we refer to the commodities in the four sectors and the stocks in the 11 industries as interconnected sector networks.

In more detail, since all series are non-stationary processes according to the unit-root test statistics, we calculate the first differenced series, i.e., the daily returns of the index, by

$$r_{i,t}=\frac{P_{i,t}-P_{i,t-1}}{P_{i,t-1}}$$ (18)

**Fig 1:**
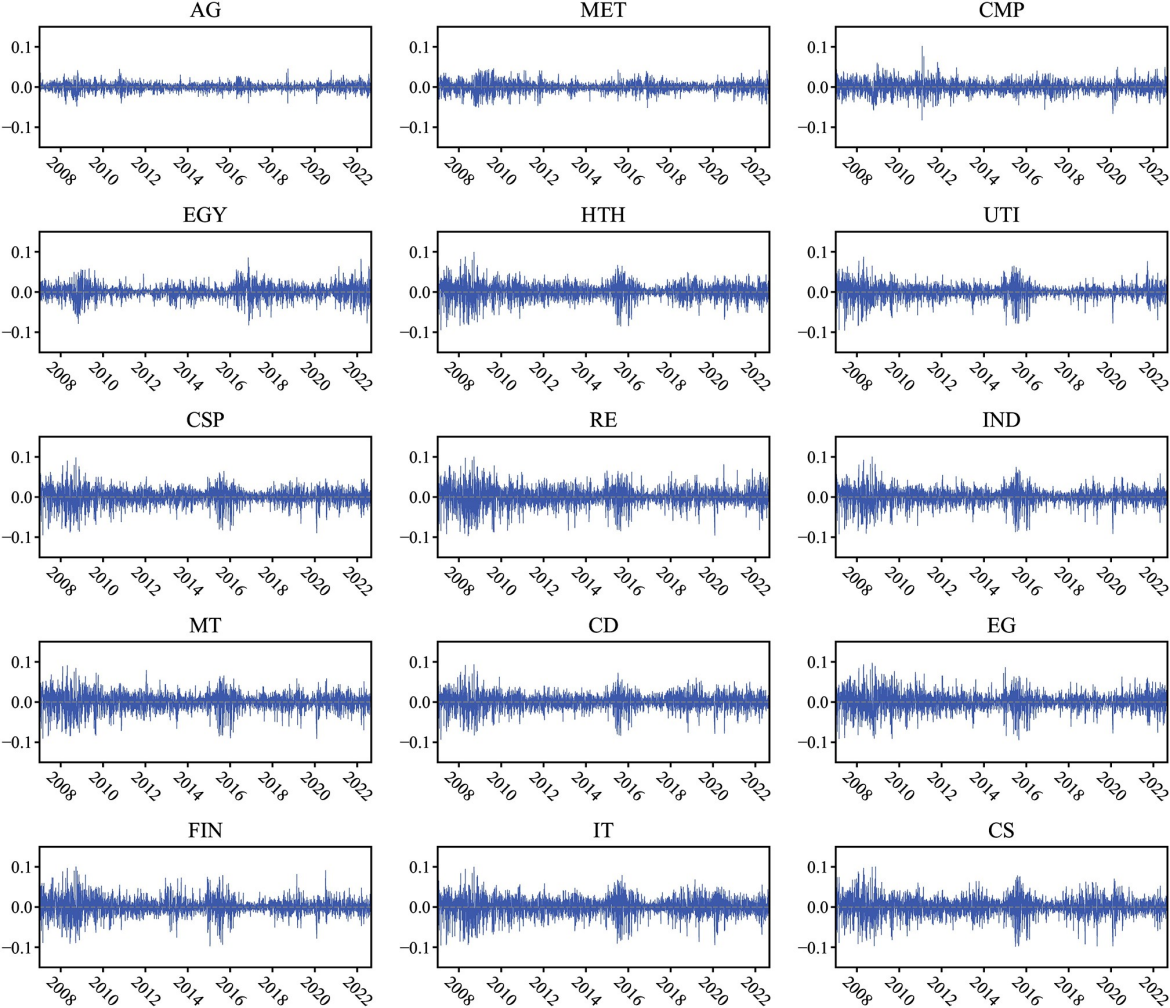
Dynamics of full-sample return.

Where *r*_*i*,*t*_ and *P*_*i*,*t*_ denote the daily return in percentage and the closing price of index i on day t, respectively. Fig 1 illustrates the daily return series of the index from February 2007 to July 2022. It shows that the dispersion of returns increased during the 2008 financial crisis, the 2015 China stock market crash, the COVID-19 breakout, and the Russia-Ukraine war.

To explore the asymmetry of return spillovers between the commodity and stocks at the sector level in China, we decompose the aggregate returns into positive and negative returns:

$$r_{i,t}^{+}=D_{i,t}\cdot r_{i,t},$$ (19)

$$r_{i,t}^{-}=(1-D_{i,t})\cdot r_{i,t},$$ (20)

$$D_{i,t}=\{\begin{array}{cc} 0, & r_{i,t}<0\\ 1, & r_{i,t}\geq 0 \end{array}.$$ (21)

Where $r_{i,t}^{+}$ and $r_{i,t}^{-}$ denote the index’s positive and negative daily returns, respectively. It represents the asymmetric response of asset returns to good and bad information.

Table 1 presents the descriptive statistics of price returns for the four sectoral commodities and 11 industry stocks. Throughout the study period, all sectors had positive average annualized returns. However, it is important to note that commodity futures generally yield lower returns than stocks, primarily due to the stricter price limit orders in the Chinese commodity market compared to the stock market. Among the sectoral commodities, energy (EGY) futures demonstrate the highest average returns, while chemical products (CMP) futures exhibit the lowest average returns. The consumer discretionary (CD) sector records the highest average returns within the industry stocks, while the energy (EG) sector displays the lowest. Interestingly, the returns ranking within the energy sector varies across the two markets. Notably, all returns, except for the financial (FIN) sector, exhibit negative skewness. Unlike the U.S. commodity market, the skewness of the Chinese commodity market demonstrates a distinct monotonic and asymmetric pattern, suggesting the presence of potential arbitrage opportunities. Moreover, the kurtosis of all returns is three times greater than the normal distribution, supporting the results of Jarque and Bera’s [70] normality test statistic, which confirms the significant departure from normality in all series. Finally, all series exhibit significant stationarity based on the Elliott-Rothenberg-Stock (ERS) unit root testing results. The weighted portmanteau test of Fisher and Gallagher [71] demonstrates significant autocorrelation in all return series and their squares. These tests validate the applicability of the TVP-VAR model for measuring sample connectedness.

**Table 1:** Summary descriptive statistics.

	AG	MET	CMP	EGY	HTH	UTI	CSP	RE	IND	MT	CD	EG	FIN	IT	CS
Mean	0.017	0.009	0.005	0.04	0.066	0.029	0.047	0.039	0.036	0.036	0.072	0.025	0.029	0.054	0.044
Variance	0.74***	1.41***	1.93***	2.33***	3.53***	2.83***	3.45***	4.71***	3.48***	3.98***	3.29***	4.17***	3.64***	4.67***	4.56***
Skewness	-0.14*** (0.000)	-0.21*** (0.000)	-0.07* (0.085)	-0.19*** (0.000)	-0.42*** (0.000)	-0.59*** (0.000)	-0.56*** (0.000)	-0.25*** (0.000)	-0.53*** (0.000)	-0.50*** (0.000)	-0.35*** (0.000)	-0.17*** (0.000)	0.09** (0.020)	-0.48*** (0.000)	-0.33*** (0.000)
Kurtosis	2.71*** (0.000)	2.16*** (0.000)	2.58*** (0.000)	3.00*** (0.000)	2.60*** (0.000)	4.21*** (0.000)	3.04*** (0.000)	2.32*** (0.000)	3.44*** (0.000)	2.47*** (0.000)	2.64*** (0.000)	2.62*** (0.000)	3.79*** (0.000)	2.06*** (0.000)	2.69*** (0.000)
JB	1175*** (0.000)	766*** (0.000)	1060*** (0.000)	1452 *** (0.000)	1183 *** (0.000)	3024 *** (0.000)	1660 *** (0.000)	895*** (0.000)	2056*** (0.000)	1127*** (0.000)	1188*** (0.000)	1107*** (0.000)	2283*** (0.000)	820*** (0.000)	1221*** (0.000)
ERS	-11.74*** (0.000)	-6.30*** (0.000)	-6.38*** (0.000)	-4.47*** (0.000)	-7.24*** (0.000)	-3.53*** (0.000)	-9.32*** (0.000)	-20.76*** (0.000)	-6.61*** (0.000)	-13.01*** (0.000)	-11.68*** (0.000)	-19.99*** (0.000)	-3.95*** (0.000)	-8.57*** (0.000)	-14.46*** (0.000)
Q(20)	24.30*** (0.002)	49.41*** (0.000)	36.20*** (0.000)	50.23*** (0.000)	47.33*** (0.000)	39.86*** (0.000)	32.03*** (0.000)	26.39*** (0.001)	38.94*** (0.000)	39.77*** (0.000)	36.37*** (0.000)	24.04*** (0.003)	31.56*** (0.000)	22.68*** (0.005)	25.77*** (0.001)
Q^2^(20)	788*** (0.000)	1949*** (0.000)	541*** (0.000)	1422*** (0.000)	1074*** (0.000)	1883*** (0.000)	1145*** (0.000)	952*** (0.000)	1419*** (0.000)	1103*** (0.000)	894*** (0.000)	747*** (0.000)	1065*** (0.000)	1081*** (0.000)	982 *** (0.000)

This table presents descriptive statistics across China’s commodity sectors and industry stocks, including Agriculture (AG), Metals (MET), Chemical Products (CMP), Energy (ERG), Energy (EG), Materials (MT), Industrials (IND), Consumer Discretionary (CD), Consumer Staples (CSP), Healthcare (HTH), Financials (FIN), Real Estate (RE), Information Technology (IT), Communication Services (CS), and Utility (UTI) sector. JB denotes the Jarque-Bera normality statistic (Jarque and Bera [70]) tests; ERS denotes the Elliott et al. [72] unit-root test; Q(20) and Q^2^(20) refer to weighted portmanteau tests of Fisher and Gallagher [71].

***, **, * denote significance at 1%, 5%, 10% significance level respectively.

### 4.2 Preliminary analysis

**Fig 2:**
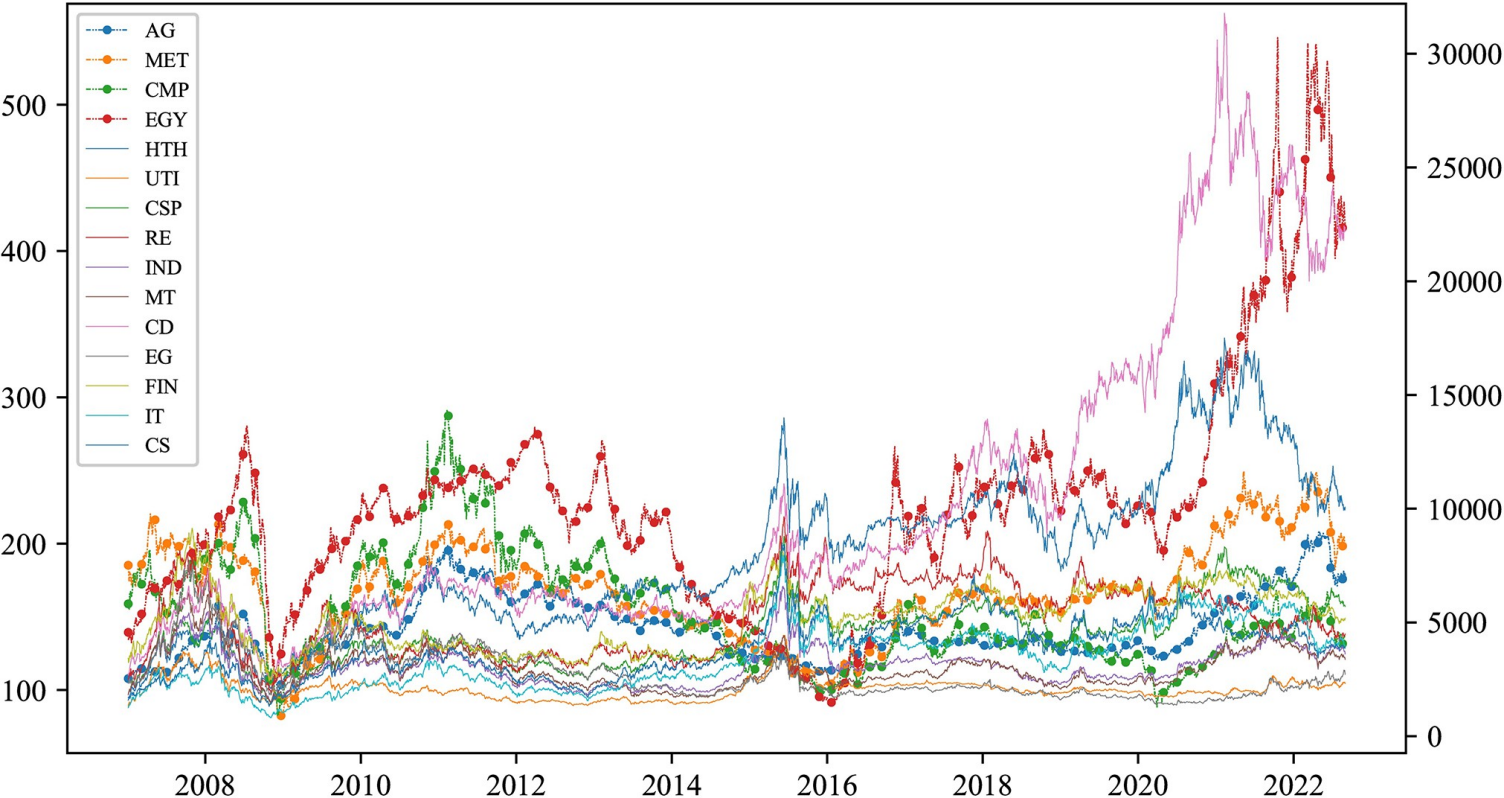
Dynamics of full-sample price indices.

We begin with an examination of the price series trends across all sectors. Fig 2 shows the time-series price of sectoral commodities and industry stock indices prices throughout the study period. The qualitative analysis reveals remarkable similarities in the trend characteristics of index prices across sectors, accompanied by significant fluctuations within the observed timeframe. Initially, all sectors showed an upward price trend, followed by a pronounced decline in response to the 2008 financial crisis. Prices bottomed out in early 2009. Subsequently, from the second half of 2009, both markets entered a recovery phase stimulated by the Chinese government’s stimulus package. However, a four-year downturn began after 2011, leading to a distinct divergence in the price performance of sectoral commodities and stocks before the Chinese stock market crash in 2015. Following the containment of the COVID-19 pandemic in China, a synchronized upward trend in the prices of the two market sectors emerged. The synchronized upward trend observed in the consumer discretionary (CD) sector and energy (EGY) futures was particularly noteworthy, with the latter slightly lagging the former. On the other hand, agricultural (AGR) futures prices showed smoother movements relative to commodities and stocks in other sectors. This observation is consistent with the strict regulation of commodity market pricing, especially in the case of agricultural prices in China.

In addition, Table 2 presents the Kendall *τ* correlation coefficients for all sector return series. T The unconditional correlation coefficients between the commodity and stock market sectors range from 0.1 to 0.714. It is noteworthy, however, that the correlation coefficients between sectoral commodities and industry stocks are generally below 0.3, lower than those between stocks in each sector and commodities in each sector. This finding is consistent with the characteristics of the Chinese market, where the level of financialization of commodities is relatively low, resulting in lower correlations between cross-market than within-market sectors. Table 2 shows a correlation coefficient of 0.251 between metals (MET) futures and energy (EG) stock sector, indicating a moderate positive correlation. In contrast, the correlation coefficient between Healthcare (HTH) and Agriculture (AG) futures is only 0.1, suggesting a weak relationship between these two sectors. These variations in the cross-sector pairwise relationships between the commodity and stock markets provide initial evidence of the heterogeneous nature of interdependencies within the Chinese financial market.

**Table 2:** Pairwise unconditional correlations between the sectoral commodities and industry stocks.

	AG	MET	CMP	EGY	HTH	UTI	CSP	RE	IND	MT	CD	EG	FIN	IT	CS
AG	1.00														
MET	0.32	1.00													
CMP	0.38	0.48	1.00												
EGY	0.27	0.41	0.40	1.00											
HTH	0.10	0.14	0.14	0.08	1.00										
UTI	0.13	0.16	0.16	0.12	0.45	1.00									
CSP	0.14	0.19	0.18	0.13	0.61	0.56	1.00								
RE	0.12	0.15	0.15	0.10	0.38	0.49	0.52	1.00							
IND	0.14	0.20	0.19	0.14	0.56	0.63	0.71	0.51	1.00						
MT	0.16	0.26	0.23	0.18	0.50	0.57	0.64	0.49	0.70	1.00					
CD	0.13	0.17	0.16	0.10	0.61	0.46	0.62	0.41	0.55	0.51	1.00				
EG	0.18	0.25	0.25	0.22	0.36	0.52	0.49	0.46	0.55	0.62	0.41	1.00			
FIN	0.15	0.20	0.21	0.15	0.34	0.46	0.46	0.51	0.47	0.46	0.39	0.48	1.00		
IT	0.12	0.16	0.16	0.11	0.58	0.50	0.66	0.41	0.64	0.56	0.52	0.41	0.37	1.00	
CS	0.12	0.15	0.16	0.11	0.49	0.48	0.58	0.40	0.58	0.51	0.47	0.41	0.37	0.66	1.00

This table represents Kendall *τ* correlation coefficients matrix across China’s commodity sectors and industry stocks.

**Fig 3:**
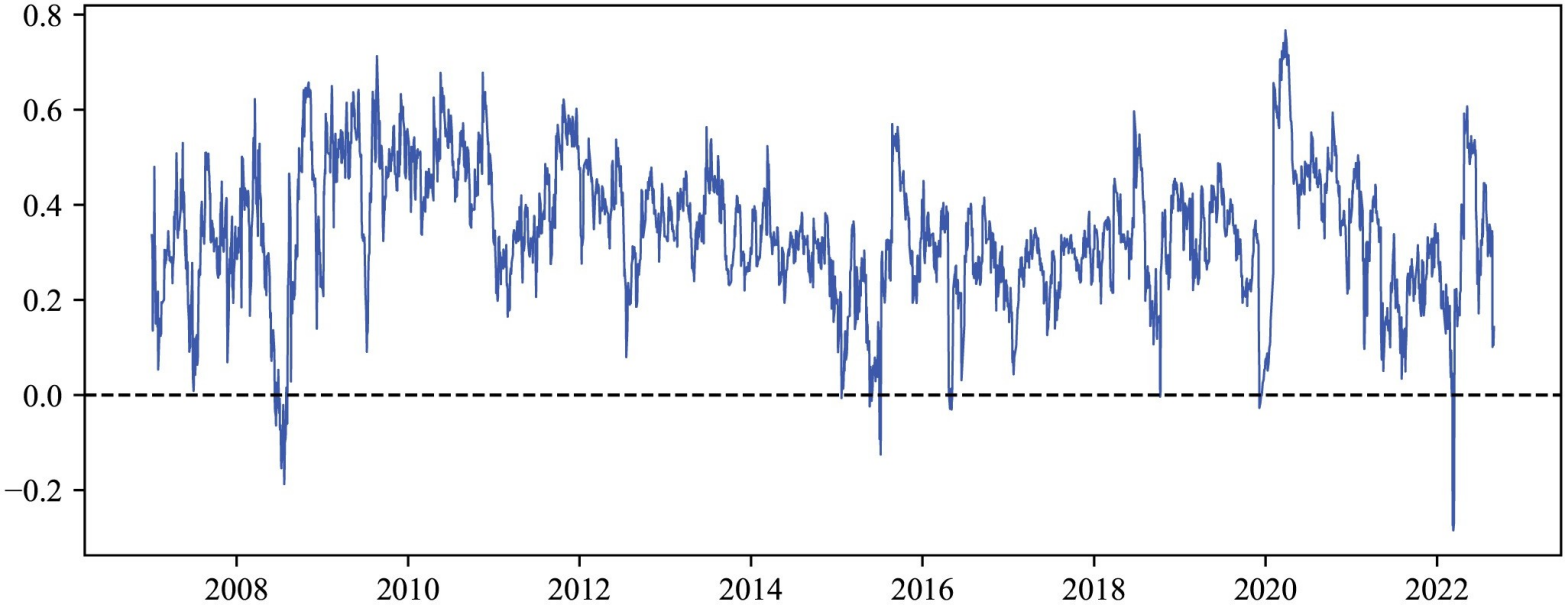
Dynamic conditional correlations. The figure shows the dynamic conditional correlations between the CSI Commodity Futures Composite Index and the CSI800 Composite Index.

Nevertheless, the correlation coefficient provides only a static description of the correlation. We provide dynamic conditional correlations in Fig 3 using the CSI Commodity Futures Composite Index and the CSI800 Composite Index based on the DCC-GARCH model. The dynamic correlation between the commodity and stock markets is evident and highly dependent on specific events and market conditions.

## 5 Empirical results and discussion

We set out both static and dynamic results of the empirical analysis of sectoral commodity and industry stock connectedness based on the TVP-VAR model. First, we provide the average Total Connectedness Index (TCI) value for the entire sample period. This provides an overview of the integrated asymmetric return spillovers and connectedness across sectoral commodities, industry stocks, and the interplay between the commodity and stock sectors. This static measure gives us a comprehensive understanding of the overall connectedness among these sectors. Next, given the vulnerability of asset prices to economic and political events, we delve into the time-varying dynamics of the TCI. This allows us to analyze the changes in sectoral connectedness in response to specific economic and political events during the sample period, which is essential for analyzing changes in the relationship between commodities and stock markets under the influence of external events. However, the TCI results can only provide a limited perspective, focusing on overall connectedness rather than the specific interactions between sectoral commodities and industry stocks. To address this, we present the results of net directional connectedness from the perspective of commodity sectors, as well as the net pairwise directional connectedness and pairwise connectedness across sectors. This analysis allows us to understand the asymmetric interactions and bilateral connectedness between sectoral commodities and industry stocks. By examining these measures, we gain a precise understanding of the formation path of connectedness between sectoral commodities and industry stocks.

### 5.1 Average connectedness measures

Based on the entire set of observations, our analysis begins with the average connectedness measures within the sector network. Tables 3 and 4 present these results, depicting the average connectedness index based on each sector’s general, positive, and negative returns.

**Table 3:** Symmetric connectedness table.

	AG	MET	CMP	EGY	HTH	UTI	CSP	RE	IND	MT	CD	EG	FIN	IT	CS	FROM
AG	49.49	8.75	12.45	7.11	1.41	2.14	2.08	1.64	2.25	2.71	2.01	2.84	1.97	1.64	1.50	50.51
MET	6.65	37.31	13.96	11.17	1.64	2.38	2.70	2.12	3.13	4.92	2.23	4.49	2.99	2.21	2.09	62.69
CMP	9.44	14.02	37.03	11.04	1.55	2.35	2.49	1.93	2.82	3.91	1.99	4.36	3.00	2.04	2.03	62.97
EGY	6.78	13.37	12.80	43.60	1.01	1.81	1.92	1.60	2.31	3.34	1.33	4.65	2.41	1.52	1.56	56.40
HTH	0.56	0.98	0.83	0.46	17.06	7.09	10.92	5.36	9.63	8.13	10.82	5.34	4.79	10.13	7.89	82.94
UTI	0.92	1.30	1.20	0.79	6.71	16.99	8.82	7.26	10.29	9.27	6.77	8.43	6.87	7.49	6.88	83.01
CSP	0.72	1.23	1.08	0.71	8.90	7.67	13.44	7.01	10.49	9.12	9.15	6.45	6.27	9.69	8.09	86.56
RE	0.84	1.30	1.15	0.79	5.84	8.14	9.22	18.86	9.21	8.66	6.41	7.68	9.19	6.51	6.19	81.14
IND	0.81	1.39	1.19	0.83	7.71	8.67	10.31	6.88	13.21	10.38	7.52	7.56	6.39	9.21	7.94	86.79
MT	0.98	2.31	1.75	1.29	6.86	8.15	9.36	6.75	10.88	13.89	7.11	9.32	6.28	8.06	7.00	86.11
CD	0.80	1.24	1.04	0.59	10.65	7.08	11.16	5.88	9.22	8.33	16.70	6.05	5.69	8.43	7.14	83.30
EG	1.33	2.62	2.48	2.36	5.36	8.53	7.76	7.02	9.28	11.03	6.09	16.55	7.62	6.01	5.98	83.45
FIN	0.99	1.83	1.73	1.17	5.52	8.01	8.60	9.17	8.85	8.30	6.57	8.65	18.89	5.80	5.91	81.11
IT	0.64	1.08	1.00	0.61	9.24	7.31	10.91	5.60	10.54	8.80	7.83	5.62	4.79	15.15	10.88	84.85
CS	0.65	1.09	1.07	0.65	7.89	7.28	9.98	5.76	9.95	8.37	7.28	6.10	5.30	11.93	16.69	83.31
TO	32.11	52.50	53.75	39.57	80.30	86.62	106.24	74.00	108.83	105.26	83.12	87.53	73.56	90.66	81.08	TCI
NET	-18.40	-10.19	-9.22	-16.83	-2.64	3.61	19.68	-7.14	22.04	19.16	-0.18	4.08	-7.55	5.82	-2.23	77.01

This table represents the averaged total connectedness index based on general returns. The results are based on the TVP-VAR model with a lag length of order one (BIC) and a generalized variance decomposition of 20-step-ahead forecast error.

**Table 4:** Asymmetric connectedness table.

Panel A. Positive return-based total connectedness index table																
+	AG	MET	CMP	EGY	HTH	UTI	CSP	RE	IND	MT	CD	EG	FIN	IT	CS	FROM
AG	53.82	7.22	9.81	5.73	1.62	2.53	2.09	1.67	2.71	2.75	2.19	2.83	1.77	1.80	1.46	46.18
MET	5.30	42.85	13.47	10.31	1.79	2.54	2.43	1.80	2.96	4.46	1.93	4.26	2.47	1.86	1.58	57.15
CMP	8.04	14.16	43.53	9.24	1.61	2.28	2.03	1.66	2.64	3.42	1.76	4.22	2.18	1.65	1.58	56.47
EGY	5.22	11.92	10.06	50.49	1.29	2.10	1.71	1.55	2.35	2.71	1.45	4.12	2.02	1.54	1.50	49.51
HTH	0.68	1.17	0.79	0.63	21.00	6.65	10.96	4.61	9.46	7.75	10.71	4.63	4.19	9.91	6.85	79.00
UTI	1.01	1.41	1.17	1.07	6.30	20.60	8.72	6.55	10.61	8.94	6.21	8.23	6.68	6.75	5.74	79.40
CSP	0.70	1.12	0.79	0.65	8.77	7.55	15.95	6.39	11.09	9.13	9.04	5.98	5.82	9.72	7.30	84.05
RE	0.82	1.22	0.98	0.82	5.35	7.77	9.01	23.84	9.06	8.14	5.68	7.31	9.16	5.71	5.12	76.16
IND	0.93	1.30	1.02	0.84	7.40	8.84	10.80	6.26	15.43	10.61	7.10	7.17	6.29	8.95	7.07	84.57
MT	0.98	2.19	1.47	1.10	6.57	7.96	9.57	6.03	11.50	16.72	6.92	9.33	6.02	7.55	6.08	83.28
CD	0.88	1.09	0.85	0.65	10.69	6.59	11.48	5.10	9.16	8.29	20.60	5.49	5.07	7.97	6.07	79.40
EG	1.27	2.51	2.16	2.27	4.87	8.66	7.53	6.53	9.31	11.32	5.63	20.44	7.47	5.10	4.93	79.56
FIN	0.91	1.54	1.24	1.08	5.00	7.89	8.20	8.97	9.10	8.02	5.84	8.48	23.54	5.20	4.97	76.46
IT	0.69	0.99	0.78	0.66	9.17	6.75	11.31	4.70	10.72	8.29	7.36	4.70	4.38	18.45	11.07	81.55
CS	0.63	0.92	0.88	0.66	7.33	6.63	9.79	4.88	9.74	7.80	6.49	5.28	4.83	12.71	21.42	78.58
TO	28.05	48.76	45.48	35.71	77.76	84.74	105.65	66.69	110.40	101.62	78.32	82.03	68.35	86.43	71.34	TCI
NET	-18.13	-8.39	-10.99	-13.80	-1.24	5.33	21.59	-9.47	25.83	18.34	-1.08	2.46	-8.11	4.89	-7.24	72.75
Panel B. Negative return-based total connectedness index table																
-	AG	MET	CMP	EGY	HTH	UTI	CSP	RE	IND	MT	CD	EG	FIN	IT	CS	FROM
AG	51.35	8.83	12.14	6.63	1.31	2.14	1.93	1.73	2.00	2.38	1.75	2.60	2.24	1.51	1.47	48.65
MET	6.89	39.53	13.72	10.28	1.53	2.74	2.58	2.28	2.95	4.17	2.00	4.00	3.36	2.04	1.92	60.47
CMP	9.61	13.72	39.30	10.10	1.34	2.59	2.38	2.21	2.70	3.41	1.75	3.86	3.19	1.88	1.94	60.70
EGY	6.30	12.25	11.80	46.30	1.09	1.90	1.95	1.90	2.13	2.94	1.33	3.97	2.88	1.60	1.65	53.70
HTH	0.54	0.89	0.74	0.46	16.85	7.41	10.64	5.86	9.35	7.96	10.67	5.53	4.98	9.89	8.21	83.15
UTI	0.87	1.55	1.36	0.78	6.87	16.13	8.87	7.45	10.12	9.26	6.77	8.40	6.95	7.55	7.06	83.87
CSP	0.68	1.22	1.08	0.71	8.74	7.90	13.15	7.30	10.37	9.10	8.87	6.58	6.57	9.51	8.21	86.85
RE	0.83	1.41	1.29	0.88	6.21	8.24	9.28	17.47	9.16	8.70	6.49	7.67	9.16	6.81	6.40	82.53
IND	0.72	1.37	1.18	0.77	7.67	8.82	10.27	7.15	12.99	10.37	7.42	7.66	6.41	9.09	8.12	87.01
MT	0.89	1.96	1.54	1.08	6.87	8.45	9.44	7.12	10.91	13.71	7.06	9.17	6.37	8.14	7.30	86.29
CD	0.75	1.12	0.92	0.60	10.56	7.25	10.88	6.22	9.01	8.21	16.54	6.13	5.83	8.42	7.56	83.46
EG	1.17	2.30	2.20	1.99	5.61	8.82	7.91	7.28	9.28	10.65	6.09	16.24	7.79	6.36	6.31	83.76
FIN	1.05	1.97	1.80	1.34	5.68	8.14	8.74	9.32	8.70	8.22	6.42	8.59	17.64	6.09	6.29	82.36
IT	0.59	1.04	0.95	0.63	9.08	7.50	10.69	6.00	10.30	8.78	7.79	5.92	5.05	14.87	10.83	85.13
CS	0.64	1.05	0.98	0.71	8.09	7.51	9.85	5.99	9.83	8.45	7.49	6.33	5.57	11.60	15.91	84.09
TO	31.54	50.67	51.70	36.96	80.64	89.42	105.41	77.82	106.80	102.60	81.91	86.41	76.36	90.50	83.29	TCI
NET	-17.12	-9.80	-9.00	-16.74	-2.51	5.55	18.56	-4.72	19.79	16.31	-1.56	2.65	-6.00	5.37	-0.80	76.80

Panels A and B represent the averaged total connectedness index based only on positive and negative returns. The results are based on the TVP-VAR model with a lag length of order one (BIC) and a generalized variance decomposition of 20-step-ahead forecast error.

Table 3 shows the symmetric averaged connectedness results. The ij-th element in this table presents the estimated contribution to the forecast error variance of sector i coming from innovations to sector j. As such, the non-diagonal elements in Table 3 represent cross-sector shocks, while the main diagonal elements illustrate heterogeneous shocks from the innovation of itself. For instance, the maximum value of 43.6% in the energy (EGY) futures column/row denotes the impact of its innovations on the forecast error variance. The remaining values in the column and row represent the shocks transmitted from energy (EGY) futures to other sectors and the shocks from other sectors to energy (EGY) futures, respectively. In addition, the non-diagonal elements’ column sums and row sums are the "To" and "From" directional connectedness indices, indicating the total directional shocks of each sector to and from other sectors. And the "NET" connectedness index row is the difference between the "TO" row and the "FROM" column, indicating the net spillover effect of each sector. Among all sectors, AG is the largest net recipient with a net connectedness index of -18.4%, while IND is the strongest net sender sector with a net connectedness index of 22.04%. Moreover, the net connectedness index for all sectoral commodities is negative throughout the study period, suggesting that commodities are net recipients of information from the stock market regarding static connectedness index performance. Therefore, it is essential to focus on shocks to sectoral commodity prices from changes in industry stock prices, which is crucial for exploring the spillover path from sectoral stocks to sectoral commodities in the process of commodity financialization, and it serves as the main research question in this paper. Finally, the total connectedness index in the lower right corner represents the share of forecast error variance caused by cross-sector innovation in the network. In other words, it measures the extent to which interactions between different sectors contribute to the overall network itself. It is approximately the grand off-diagonal column sum (or row sum) relative to the grand column sum including diagonals (or row sum including diagonals), expressed as a percentage. The TCI value of 77.01% indicates robust connectedness between sectors in this network. To examine the linkages between commodity and stock markets, we exclude the contribution of inter-industry stock connectedness to the TCI and consider only the TCI between sectoral commodities and sectoral commodities and between sectoral commodities and industry stocks, which also reaches 62.82%, indicating that China’s commodity and stock markets are closely connected and show significant financialization of commodities. In addition, the "To" connectedness index row highlights the total directional shocks of each sector to all other sectors, and the "From" connectedness index column indicates the total directional shocks from other sectors. We observe that the transmission of innovations between sectoral commodities and industry stocks is asymmetric. Sectoral commodities receive more transmission of innovations aggregate shocks from industry stocks (31.89%), and fewer aggregate shocks are transmitted to industry stocks (12.6%). Moreover, the total spillover of each sectoral commodity from other sectoral commodities and the stock market is nearly equivalent, emphasizing the significant impact of the stock market on various commodity sectors.

In Table 4, Panel A and B depict the averaged total connectedness index results only measuring positive and negative returns, respectively. The finding is similar to those presented in Table 3. In more detail, the TCI index based on positive returns (76.8%) in Panel A is larger than the TCI index based on negative returns (72.75%) in Panel B; This result indicates evidence of asymmetric connectedness with negative returns having a more significant share of innovation within the network. Comparing the net connectedness index rows for panels A and B, the results reveal that the direction of the net spillover does not change across sectors due to the difference between positive and negative return shocks; however, the intensity of the spillover changes across sectors. For example, the negative return net connectedness index for Chemical Products (CMP) futures increased by 1.99% compared to the positive return net connectedness index, while the negative return net connectedness index for Energy (EGY) futures decreased by 2.94% compared to the positive return net connectedness index. These findings illustrate the heterogeneity of asymmetric spillovers across sectors and the complexity of sectoral relationships.

**Fig 4:**
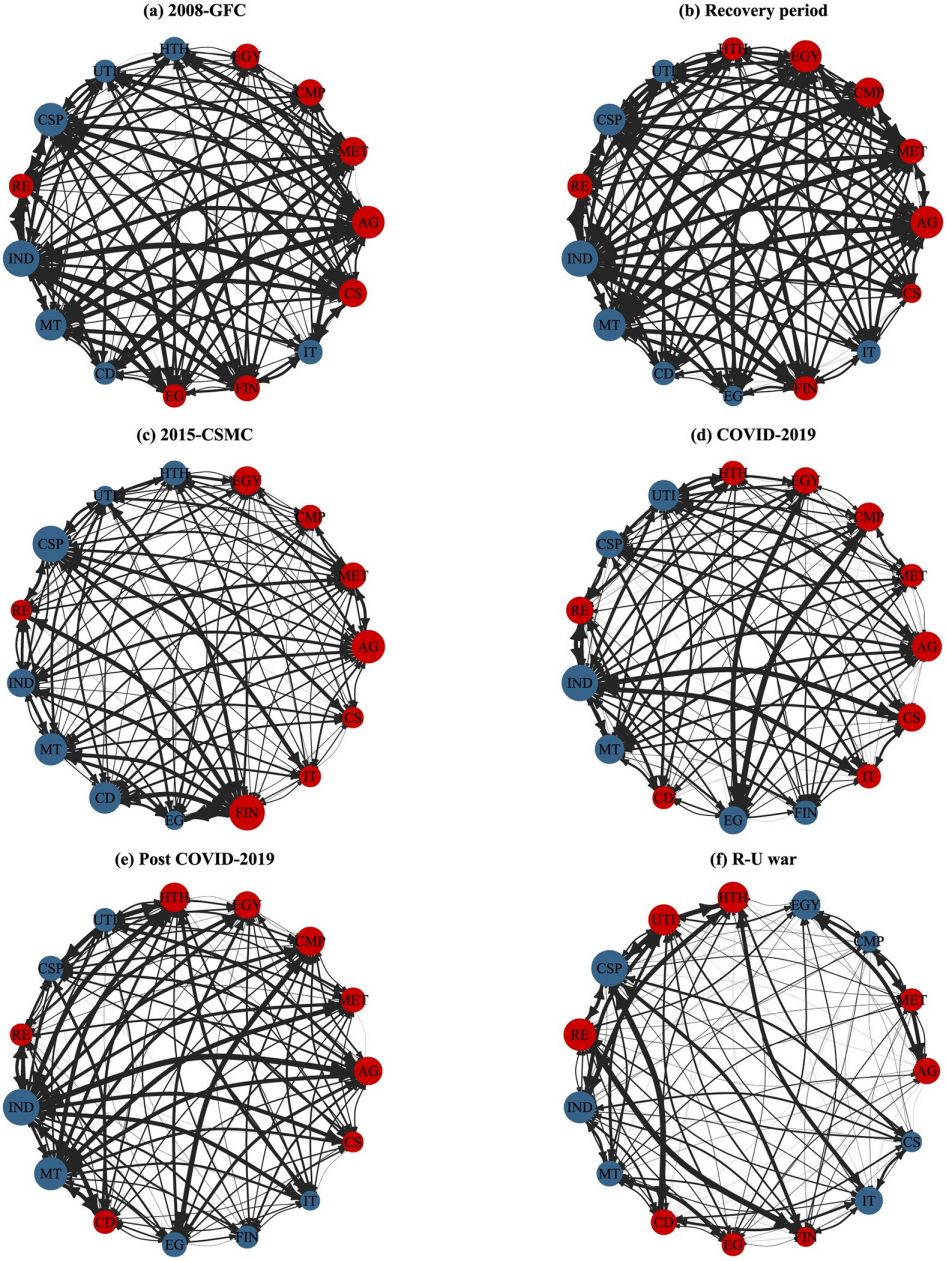
Net pairwise connectedness networks during the six notable periods. The thickness of the line and arrow reflects the size of the connectedness, the blue (red) nodes represent the transmitters (recipients), and the size of the node indicates the economic size of the link between nodes. It includes four panels: (a) depicts the period of the 2008 global financial crisis (GFC); (b) displays the post-GFC recovery period; (c) depicts the period of the 2015 Chinese stock market crisis (CSMC); (d) depicts the COVID-19 pandemic period; (e) the post-pandemic period; (f) the recent Russia-Ukraine war period.

In addition, Fig 4 displays six subgraphs (a-f) that depict networks representing the static pairwise directed connectedness between sectoral commodities and industry stocks during six significant periods: the 2008 global financial crisis (GFC), the ensuing recovery period, the 2015 Chinese stock market crisis (CSMC), the COVID-19 pandemic, the post-pandemic era, and the recent Russia-Ukraine war. This result aims to provide relevant information about the transmitter or receiver in the connectivity network and the strength of the connectivity. In the six subgraphs, the size and color of the nodes, as well as the lines between the nodes, specifically indicate the nature of the market. For example, the size of the nodes indicates the economic size of the connectedness between the pair of sectors, the red (blue) color of the nodes indicates the receivers (transmitters) in the network, and the thickness of the edges connecting the nodes to each other and the thickness of the arrows reflect the strength of the directed connections.

We find that the commodity sector remains a net recipient during key political and economic events, with only the recent outbreak of the Russia-Ukraine war, which led to an increase in global energy prices, turning the energy (EGY) and chemical products (CMP) commodity sectors into net transmitters, along with a significant increase in the intensity of spillovers across commodity sectors (Fig 4(F)). The agricultural (AG) commodities sector is consistently the largest net recipient of return spillovers among commodity sectors. Additionally, the intensity of return spillovers between commodity sectors is weaker than between commodity and stock sectors.

Furthermore, the post-crisis period shows stronger inter-sectoral connectivity for both commodities and stocks. For example, the curves of inter-nodal linkages are thicker in subgraphs 4(b) and 4(e) of Fig 4. However, inter-sectoral linkages vary during crises of different origins. For instance, during the 2008 financial crisis, which posed a global systemic risk, the correlation between nodes exhibited greater strength in Fig 4(A). Here, the industrials (IND) and consumer staples (CSP) stock sectors proved to be the largest transmitters of spillovers, while the agricultural (AG) futures emerged as the largest recipients of spillovers. During the 2015 Chinese stock market crisis (Fig 4(C)), turmoil in the stock market spread to the entire securities sector. As a result, the financial stocks sector received the largest spillover effects. During the COVID-19 pandemic outbreak, international crude oil prices were notably volatile. In Fig 4(D), the directed edges between the energy stocks node, the energy futures sector node, and the chemicals sector futures node are significantly coarsened, indicating a strong correlation between these sectors.

However, the above analysis provides only a static perspective on the potential connectedness within the sectoral commodity and industry stock networks and lacks insight into the evolving nature of commodity financialization in China. Moreover, considering the influence of major economic and political events on sector network connectedness during the sample period, adopting a time-varying dynamic analysis framework becomes crucial. Such an approach allows for a more comprehensive understanding of the asymmetric interrelationships among sectors and provides valuable insights into the dynamic nature of commodity financialization in the Chinese market.

### 5.2 Dynamic total connectedness

Over the past 20 years, the global economy has undergone significant transformations, with China emerging as the world’s largest economy and possessing the largest commodity market by trade volume. Changes in domestic and international economic and political situations have made China’s commodities market highly sensitive. The connectedness index table in the previous section only reveals potential connectedness between sectoral commodities and industry stocks. However, it does not enable observation of changes in intersectoral connectedness over time or the impact of external shocks on sector network connectedness. Constructing a time-varying dynamic analysis framework is crucial for effectively analyzing the impact of major events on sector network connectedness throughout China’s two decades of economic and financial development, providing an empirical reference for market participants.

**Fig 5:**
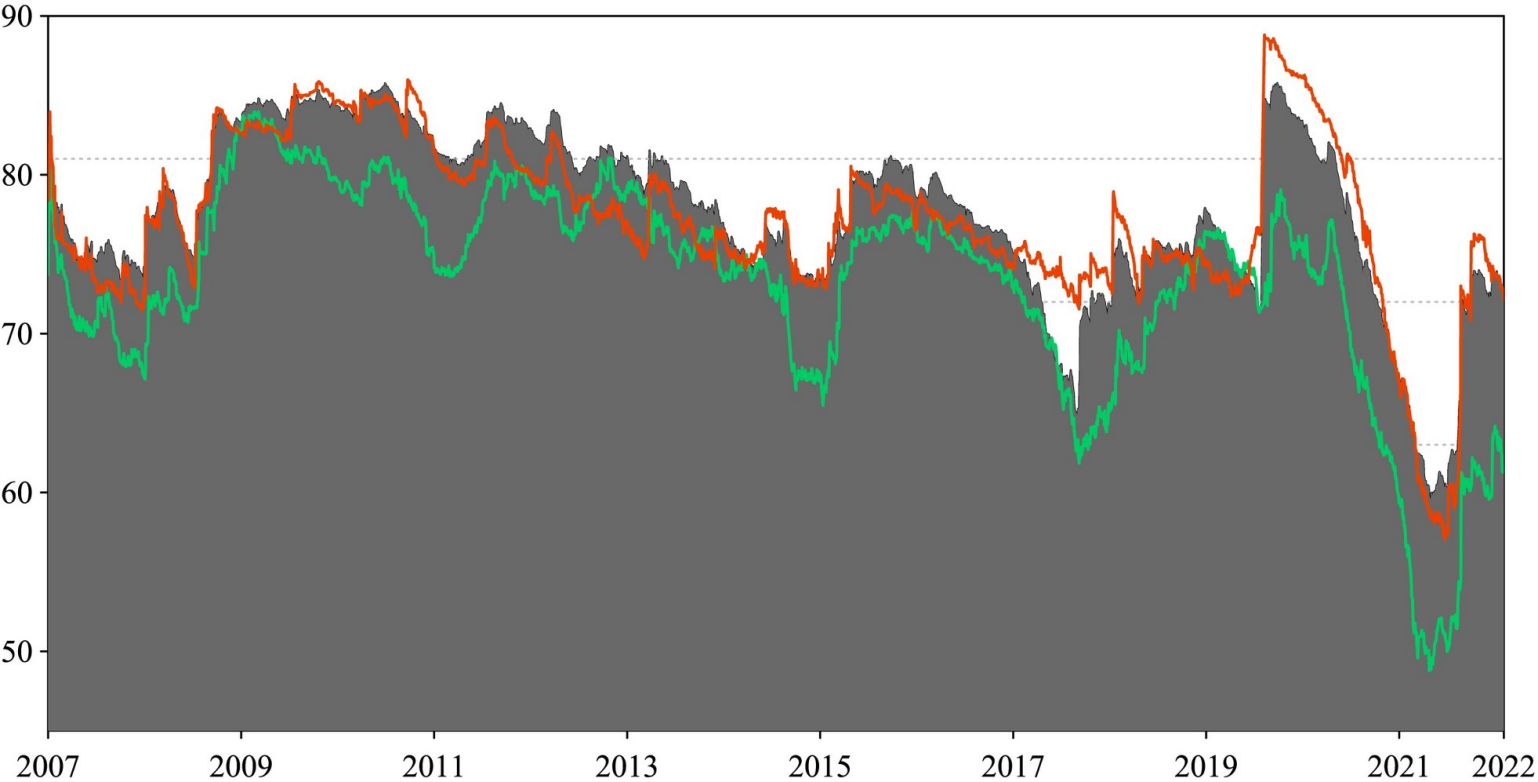
Dynamic total connectedness. This figure presents the dynamics total connectedness index (TCI) among all sectors. The shaded areas are symmetric dynamic TCI based on general returns, and the solid green and red lines refer to the asymmetric dynamic TCI based only on positive and negative returns. The results are based on the TVP-VAR model with a lag length of order one (BIC) and a 20-step-ahead generalized forecast error variance decomposition. (The latter connectedness results are the same).

**Fig 6:**
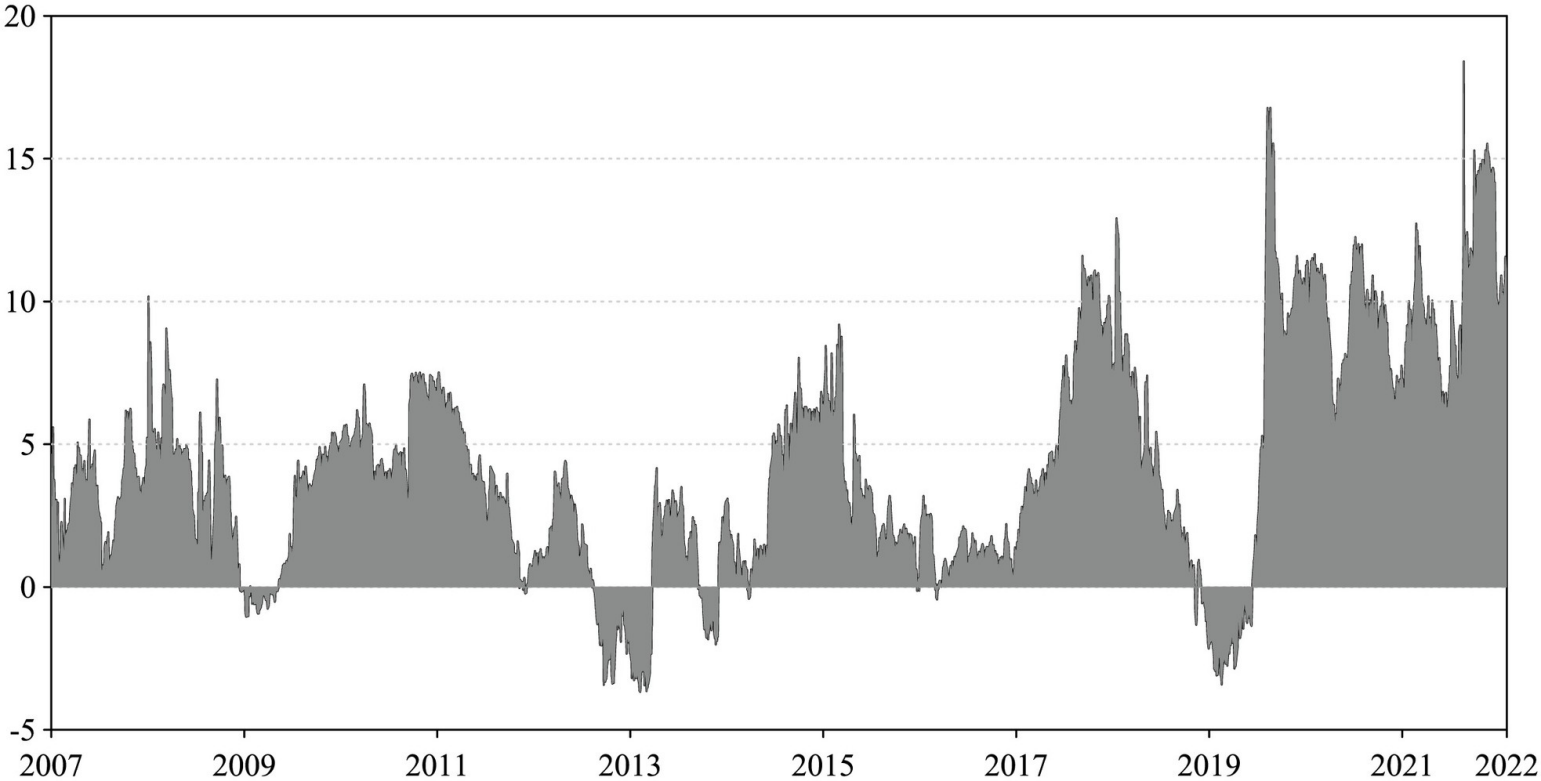
Asymmetry of dynamic total connectedness. This figure presents the difference between the dynamic total connectedness index, measured by negative and positive returns among all sectors. The results are based on the TVP-VAR model with a lag length of order one (BIC) and a generalized variance decomposition of 20-step-ahead forecast error.

We present results for dynamic total connectedness and asymmetry return spillovers in Figs 5 and 6. Fig 5 shows the temporal evolution of dynamic total connectedness between the commodity and stock market sectors, with the shaded area representing symmetric dynamic total connectedness considering both positive and negative returns. The dynamic total connectedness fluctuates between 54% and 86%, suggesting it is event-driven and cyclical. Three cycles of dynamic total connectedness are identifiable: the first spans from the sample period’s start to 2009, the second from 2010 to 2015, and the third from 2016 onward. Events like the 2008 financial crisis influenced these cycles, China’s four-trillion yuan stimulus package, "The 12th Five-Year Plan," and the 2015 stock market crisis, as well as other systemic uncertainty events such as the China-US trade dispute, the COVID-19 pandemic, and the Russo-Ukrainian war.

Fig 5 also illustrates dynamic asymmetric total connectedness, with green and red solid lines representing the dynamic asymmetric total connectedness when considering only positive and negative returns. This reveals a notable difference between positive and negative returns, with negative returns dominating the sample period. Fig 6 further emphasizes this asymmetry, demonstrating the dominance of negative returns connectedness. This dominance reflects the natural noise nature of negative shocks and that investors, especially risk-averse ones, respond more to negative than positive information. A black-swan event—the COVID-19 pandemic—in 2020 exemplified this, causing the maximum level of asymmetric connectedness as investors absorbed more negative information and reallocated their investments. This finding is consistent with the results of Shahzad et al. [50] regarding the inter-sectoral asymmetric spillover effect in the Chinese stock market during the epidemic, where they argue that bad volatility spillover shocks dominate good volatility spillover shocks.

We extend this conclusion to the inter-sector spillover (connectedness) analysis encompassing both the commodity and stock market, which sheds light on the financialization of commodities in China by revealing the connectedness between sectoral commodities and industry stocks and the influence of economic and political events on sector network connectedness. The event-driven and cyclical nature of the dynamic total connectedness also indicates that these events affect the commodity financialization process.

### 5.3 Directional connectedness

In our previous analysis of the static and dynamic total connectedness index (TCI), we found evidence of asymmetric return spillovers, with the commodity market acting as a net recipient. Shifting the focus to a sectoral commodity perspective, we investigate the connectedness of different sectors: agriculture, metals, chemical products, and energy futures with all industry stocks. This sectoral approach allowed a focused examination of the interdependence between sectoral commodities and the stock market, enabling investors to develop more effective trading strategies.

**Fig 7:**
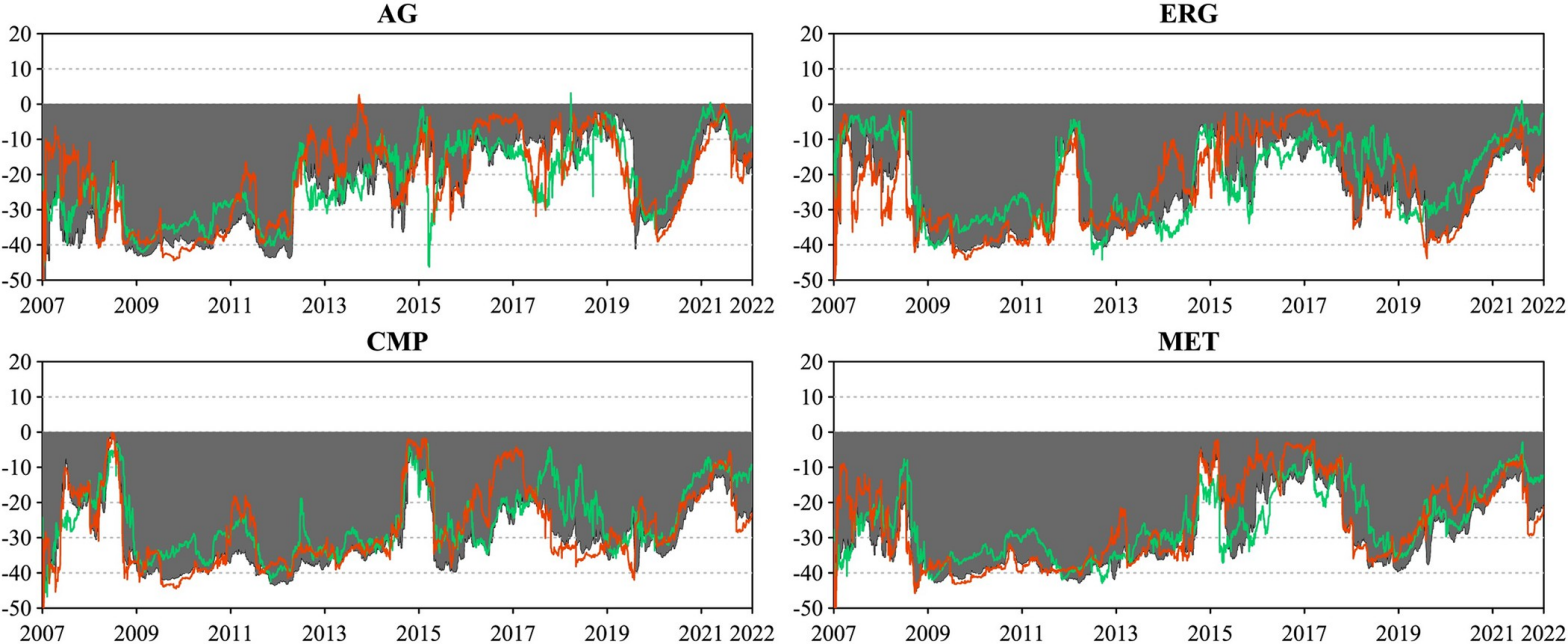
Dynamic net connectedness for each commodity sector. This figure presents the dynamic net directional connectedness index between the four commodity sectors and all industry stocks. The results are based on the TVP-VAR model with a lag length of order one (BIC) and a generalized variance decomposition of 20-step-ahead forecast error.

**Fig 8:**
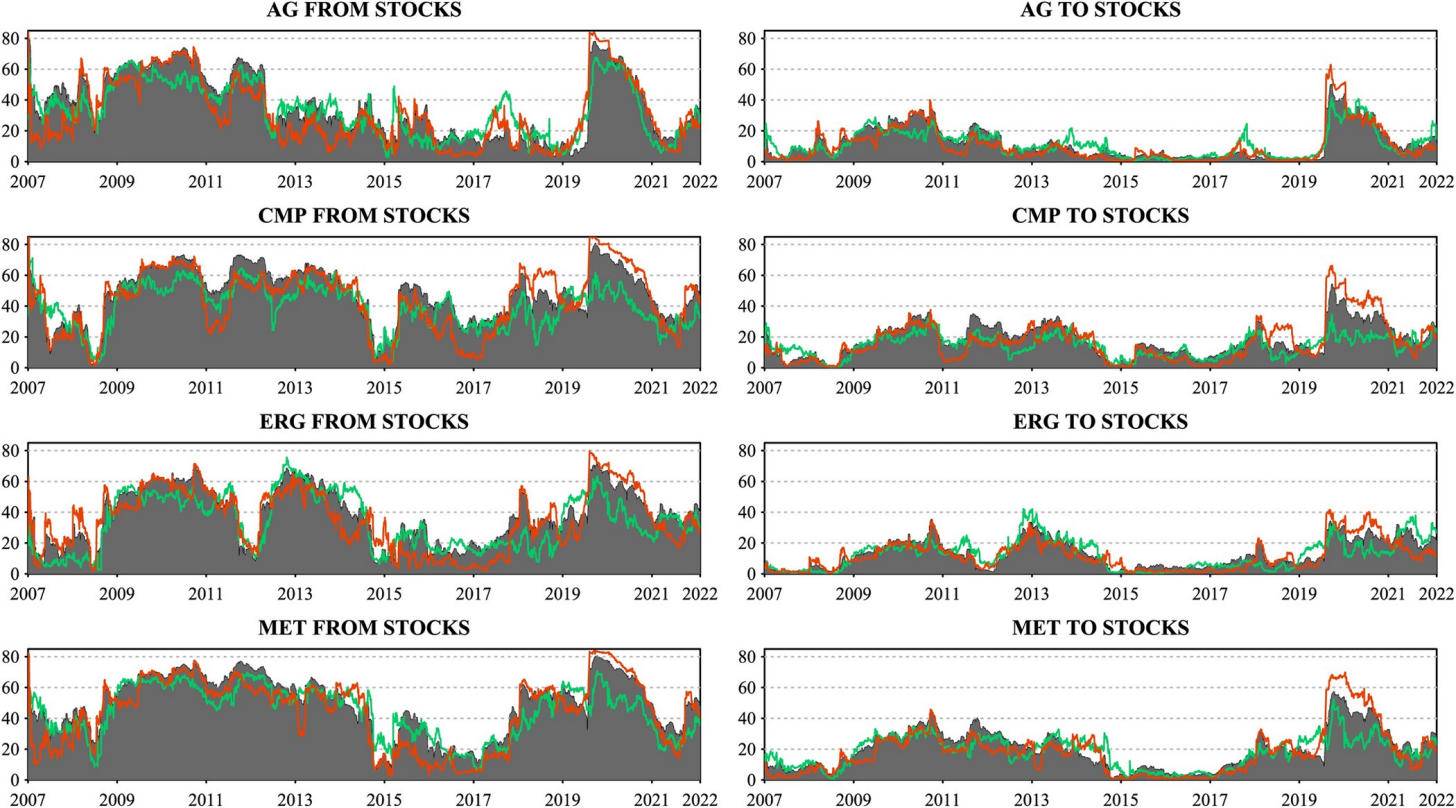
Directional connectedness for each commodity sector. This figure presents the four commodity sectors’ dynamic net directional connectedness index to and from all industry stocks. The results are based on the TVP-VAR model with a lag length of order one (BIC) and a generalized variance decomposition of 20-step-ahead forecast error.

We present the dynamic directional connectedness between sectoral commodities and all industry stocks and their asymmetric spillover variation in Figs 7 and 8. This will highlight the differences in the spillover patterns and their changing intensity and direction between sectoral commodities and stock markets.

In Fig 7, we show the net directional connectedness of the four commodity sectors, where positive and negative values indicate net transmitters and recipients, respectively. All sectoral commodities were net recipients of stock market return spillovers throughout the study period, consistent with previous results. However, the asymmetry in return spillovers across sectoral commodities differs from the total dynamic connectedness (TCI), rather than being dominated by negative returns, and the asymmetric return spillovers across sectoral commodities are heterogeneous over time and driven by political, economic, and other events during the study period. This pattern notably occurred during the 2008 financial crisis and the 2015 China stock market crash, with the connectedness index decreasing and the connectedness gap between positive and negative returns shrinking remarkably. Despite sectoral commodities being net recipients of spillovers, they did not experience increased negative spillovers during the period of financial turbulence. In contrast, such events led to capital flows from stock to commodity markets, which reduced the connectedness of returns and resulted in positive returns dominating the asymmetric connectedness. This finding suggests that incorporating commodities in a diversified market portfolio is beneficial in China even during turbulent times. Yet, the COVID-19 pandemic painted a different picture, with the entire capital market experiencing an unprecedented shock and panic spread rapidly in both the commodity and stock markets, an increase in the net directional connectedness index, and a shift in asymmetric connectedness dominated by negative return spillovers. Similarly, the spike in international energy and shipping prices following the Russia-Ukraine war in early 2022 reinforced asymmetric return spillovers across all commodity sectors, triggering a shift toward positive return-dominated spillovers.

In addition, Fig 8 estimates the bidirectional connectedness indices, capturing the directional spillover FROM and TO the stock market for each sectoral commodity. The four left-hand subplots present each commodity sector’s dynamics and directional connectedness from industry stocks, corresponding to the “FROM” columns in Table 4. Since all sectoral commodities are net recipients of stock market return spillovers, the asymmetric return spillovers of this result are similar to the net directional connectedness of Fig 7, but their symmetry connectedness differs. For instance, agriculture (AGR) futures, possibly reflecting China’s "12th Five-Year Plan" food security policy that calls for stable prices for agricultural products, showed weaker shocks compared to other sectors before the COVID-19 pandemic, with around 20% (FROM) directional connectedness. In contrast, the chemical products (CMP) futures reached about 40% during the same period. These results confirmed the crucial role of event-driven factors on the dynamic connectedness between commodity and stock markets from a sectoral commodity perspective.

In contrast to sectoral commodity spillovers from the stock market, sectoral commodity spillovers to the stock market shown in the four right-hand subplots of Fig 8 are considerably less. Over the period studied, the metals sector, which has the highest degree of spillover to the stock market, has an average value of only 16.37%, indicating that the impact of the commodity market on the stock market through spillover effects is very limited. Therefore, we argue that the path of commodity financialization formation represented by the connectedness of China’s commodity and stock markets relies mainly on the net return spillover from the stock market to the commodity market. Moreover, regarding the asymmetric return spillovers from sectoral commodities to stock markets, after 2012, the positive return dominated the asymmetric TO directional connectedness until 2015, except for the chemicals (CMP) futures. Especially evident during the Russia-Ukraine war, which caused a spike in global energy and food prices and subsequently affected the stock market, the TO directional connectedness from the agricultural (AG) and energy (EGY) futures to stock markets became dominated by positive returns.

Our analysis underscores the importance of considering sectoral commodities when studying spillover effects or measuring connectedness between Chinese commodities and stock markets, as the absence of significant commodity investment vehicles in the Chinese financial market and the prevalence of retail investors in the futures market. There is no such thing in China as "commodity financialization" in the U.S., where index traders influence price formation, leading to stronger correlations between commodity prices and traditional asset markets. Net information spillovers from stock markets have mainly driven the increased connectedness between commodity and stock markets in China. It has been subject to various political and economic events, resulting in significant differences in the connectedness across sectoral commodities and stock markets. Recognizing the distinct characteristics of China’s financial market, particularly its lack of true commodity financialization, can provide valuable insights for investors and policymakers seeking to navigate risks and identify opportunities for portfolio diversification.

### 5.4 Pairwise connectedness

In the interconnectedness of sectoral commodities and the stock market, not only do the sectoral commodities act as net recipients of stock market return spillovers, but the different sectoral commodities also exhibit different time-varying dynamics from the stock market. To delve deeper into this dynamic interconnectedness and to reinforce the findings presented in the previous sections, we now shift our focus towards a more granular analysis, examining the pairwise connectedness between the four commodity sectors—agriculture, metals, chemical products, and energy futures—and the 11 distinct industry stocks. This aim is to provide a detailed investigation of the directional connectedness emanating to and from the individual industry stocks for each commodity sector, emphasizing the asymmetric returns spillover variations and the time-varying characteristics of the spillover strength of these sectoral commodities as either transmitters or recipients within the network, and how different economic conditions and events affect these sectoral dynamics. To enhance understanding of the dynamic interconnectedness across sectoral commodities and stocks in China’s financial market.

**Fig 9:**
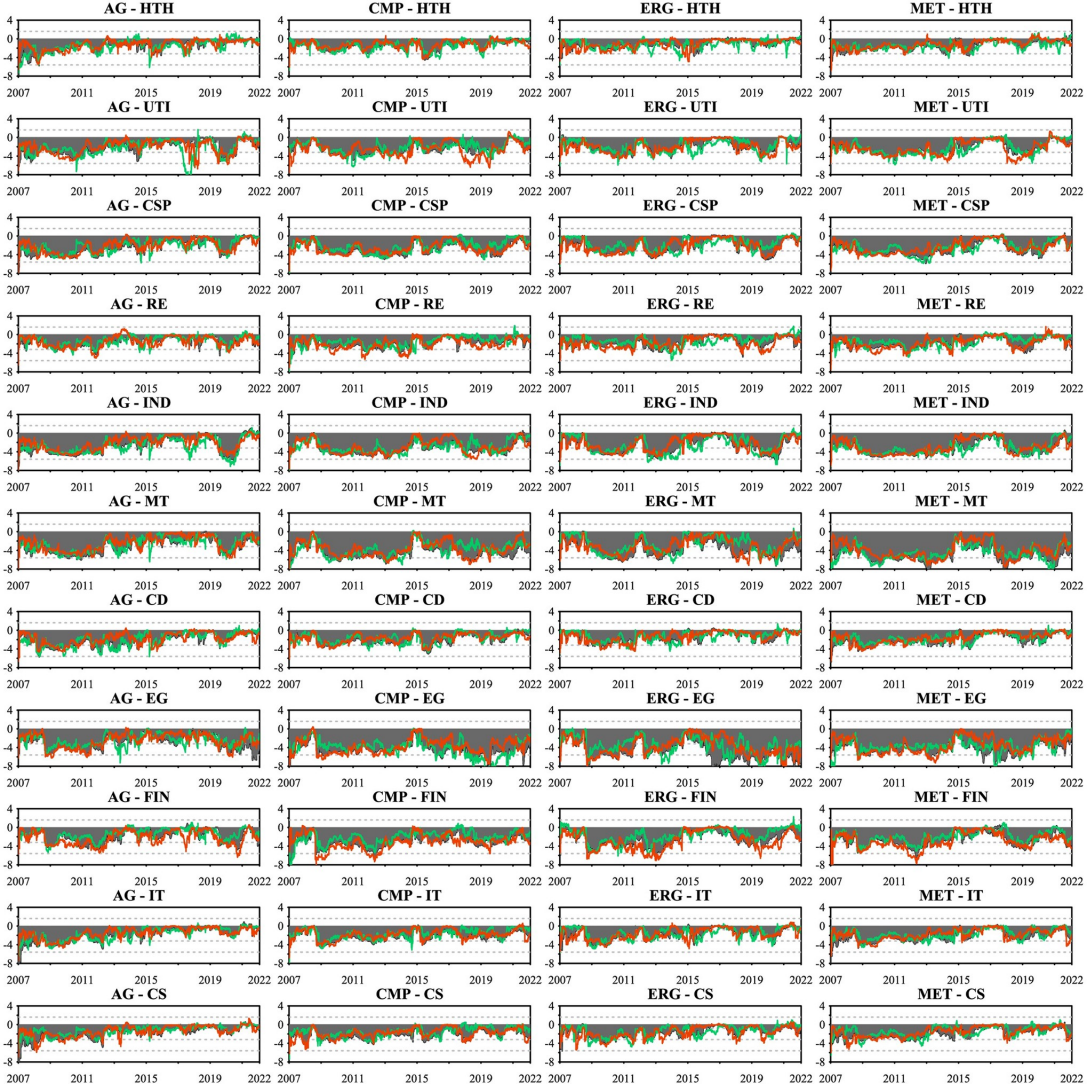
Dynamic net pairwise directional connectedness. This figure presents the dynamic net pairwise directional connectedness index between 4 commodity sectors and 11 industry stocks. The results are based on the TVP-VAR model with a lag length of order one (BIC) and a generalized variance decomposition of 20-step-ahead forecast error.

We begin with the dynamic net pairwise directional connectedness between four commodity sectors and 11 industry stocks shown in Fig 9, where positive values correspond to net transmitters and negative values to net recipients in the bilateral return spillovers relationship. Apparently, all sectoral commodities remain persistent net recipients of return spillovers from the eleven industry stocks throughout the period under study. In more detail, we find the three industry stocks that have contributed the most substantial return spillovers to the four sectoral commodities as being the Materials (MT), Energy (EG), and Industrials (IND) sectors. This correlation may arise because the companies included in these three sectors are typically involved in producing or processing commodities, leading to a tight relationship between these industry stocks and commodities. In addition, the stocks from the Financials (FIN) and Consumer Discretionary (CSP) sectors rank fourth and fifth, respectively, as net return spillover sources to all sectoral commodities except agricultural futures. Consumer Discretionary (CSP) and Consumer Staples (CS) rank fourth and fifth, respectively, in terms of net return spillovers to the Agricultural (AGR) commodity sector. These findings unequivocally demonstrate the connectedness between sectoral commodities and industry stocks established through production relationships or the degree of demand intensity.

**Fig 10:**
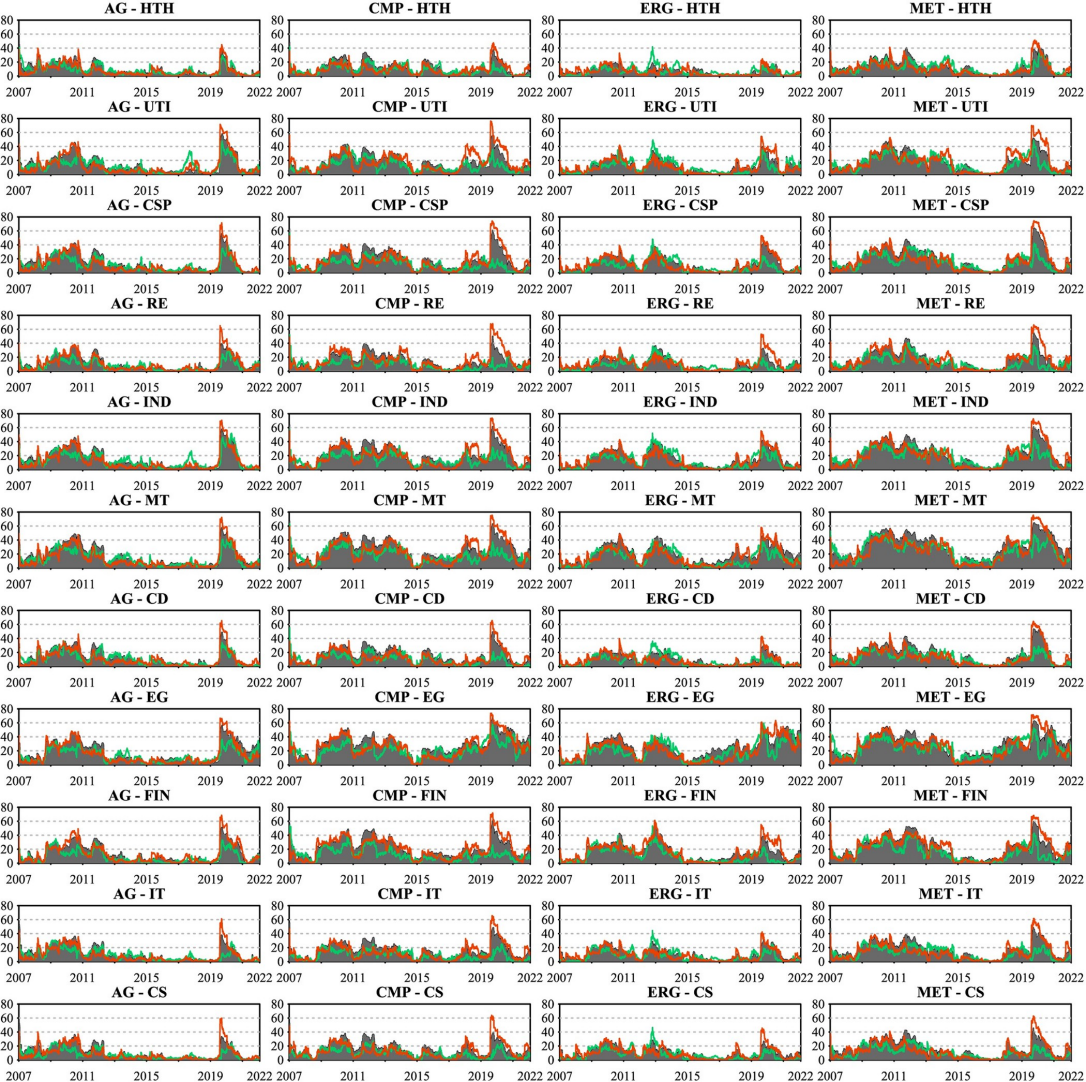
Dynamic pairwise connectedness. This figure presents the dynamic pairwise connectedness index between 4 commodity sectors and 11 industry stocks. The results are based on the TVP-VAR model with a lag length of order one (BIC) and a generalized variance decomposition of 20-step-ahead forecast error.

Fig 10 presents the dynamic pairwise connectedness. Unlike the net directional connectedness that reflects the transmitter or receiver information within the connectedness of various sectors, this metric, based on Gabauer’s [18] decomposition of the Total Connectedness Index (TCI), ranges between [0,1]. It emphasizes the comprehensive bilateral interconnectedness and asymmetry of return spillovers between sectoral commodities and industry stocks, providing a more robust examination of the findings in the previous sections. The results in Fig 10 are consistent with earlier findings, confirming a significant discrepancy between positive return spillovers and negative return spillovers across sector commodities and industry stocks, which become more pronounced since the onset of 2020. We observed that following the outbreak of the COVID-19 pandemic, the gap between the negative and positive return spillovers of sectoral commodities and industry stocks peaked within the study period, indicating that the impact of negative shocks was maximized during the COVID-19 period. This observation supports the argument by Corbet et al. [73] regarding the role of COVID-19 as a "black swan" event in establishing information asymmetry in the Chinese market.

Further compounded by the inherent complexities of modern financial markets [17] and the heterogeneous sensitivity of investors to similar market news [74], this provides compelling evidence for asymmetric return spillovers during the COVID-19 pandemic. Similarly, these asymmetries were confirmed following the outbreak of the Russia-Ukraine war towards the end of the sample period. Global energy security was threatened, leading to a rise in international energy prices. As a result, the asymmetrical spillover effects dominated by positive return spillovers between sectoral commodities and the energy (EG) sector stocks became more pronounced. Furthermore, the results of symmetric connectedness depicted in the shaded area of Fig 10 are also consistent with the findings of symmetric net directional connectedness in Fig 9. These results present the dynamic relationship between the two market sectors over time.

### 5.5 Robustness tests

**Fig 11:**
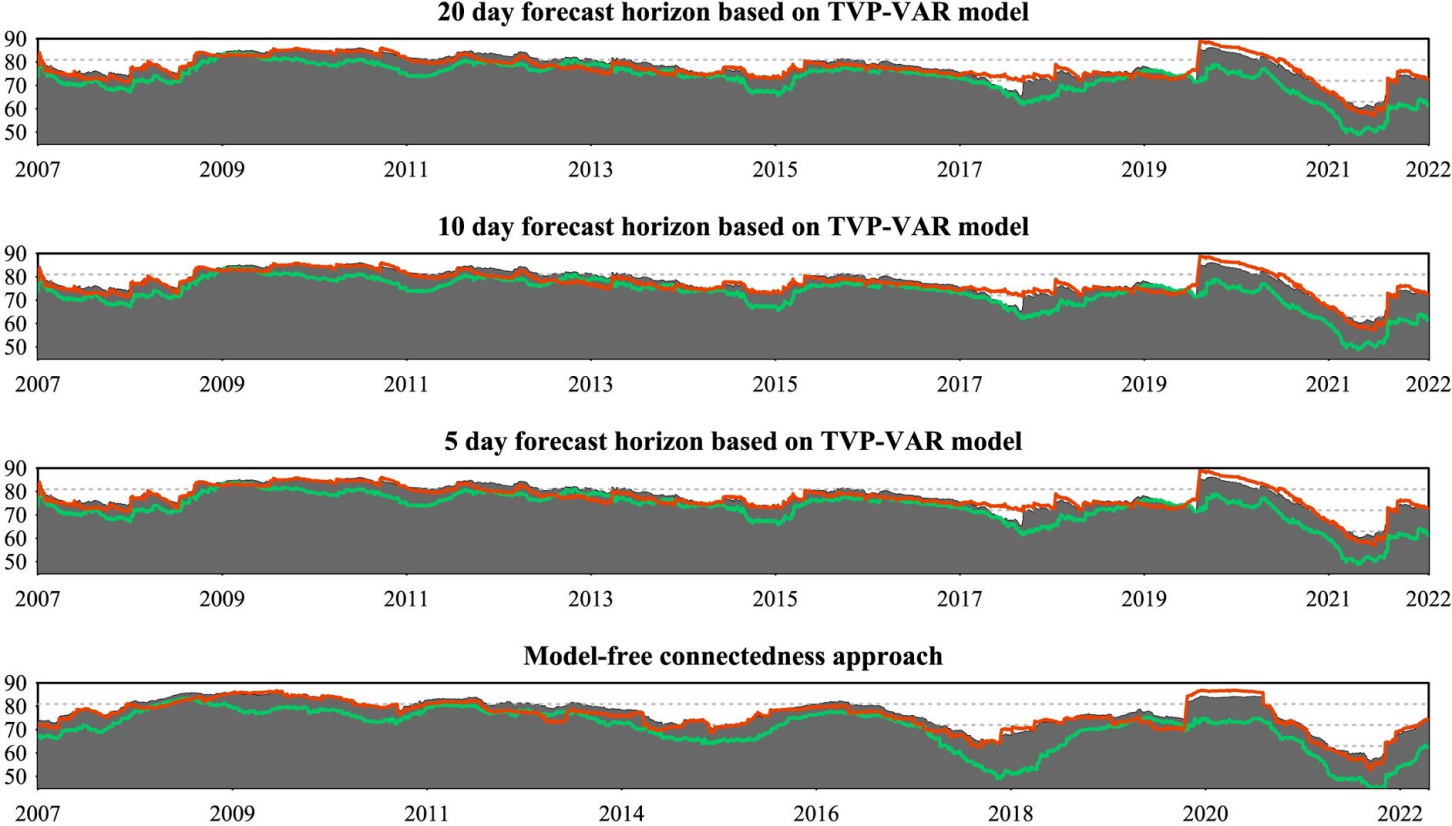
Robustness test of total connectedness indices. This figure presents a robustness test for the total connectedness of asymmetric returns with four panels. (a) 20-day forecast horizon based on the TVP-VAR model; (b) 10-day forecast horizon based on the TVP-VAR model; (c) 5-day forecast horizon based on the TVP-VAR model; (d) model-free connectedness approach with a 200-day rolling window.

Fig 11 presents the robustness test of the total connectedness index for overall returns, positive and negative returns. We estimate the total connectedness index through two different approaches: firstly, employing the TVP-VAR model with a generalized variance decomposition, considering H-step-ahead forecast errors with varying horizons of 20, 10, and 5 days, and secondly, utilizing the model-free connectedness approach with 200-day rolling windows [75]. The results show a consistent pattern across these metrics, regardless of the forecast days chosen and the model selection. This underscores the robust nature of the total connectedness index and confirms its stability across TVP-VAR model selection and forecast horizon adjustment.

### 5.6 Portfolio analysis

Our preceding empirical findings, which hold significant implications for portfolio diversification and risk management strategies, establish the existence of return spillovers between sectoral commodities and industry stocks. This section delves into the practical application of our research theme. We aim to enhance market management and portfolio construction by analyzing the asymmetry in return spillovers and constructing portfolios based on three multivariate portfolio construction methods: the maximum variance portfolio (MVP) method, the minimum correlation portfolio (MCP) method and the minimum connectedness portfolio (MCoP) method. The objective here is to assess the performance of minimize connectedness portfolios, thereby better-capturing asymmetries in return spillovers. This is particularly valuable for investors with asymmetric risk preferences.

**Fig 12:**
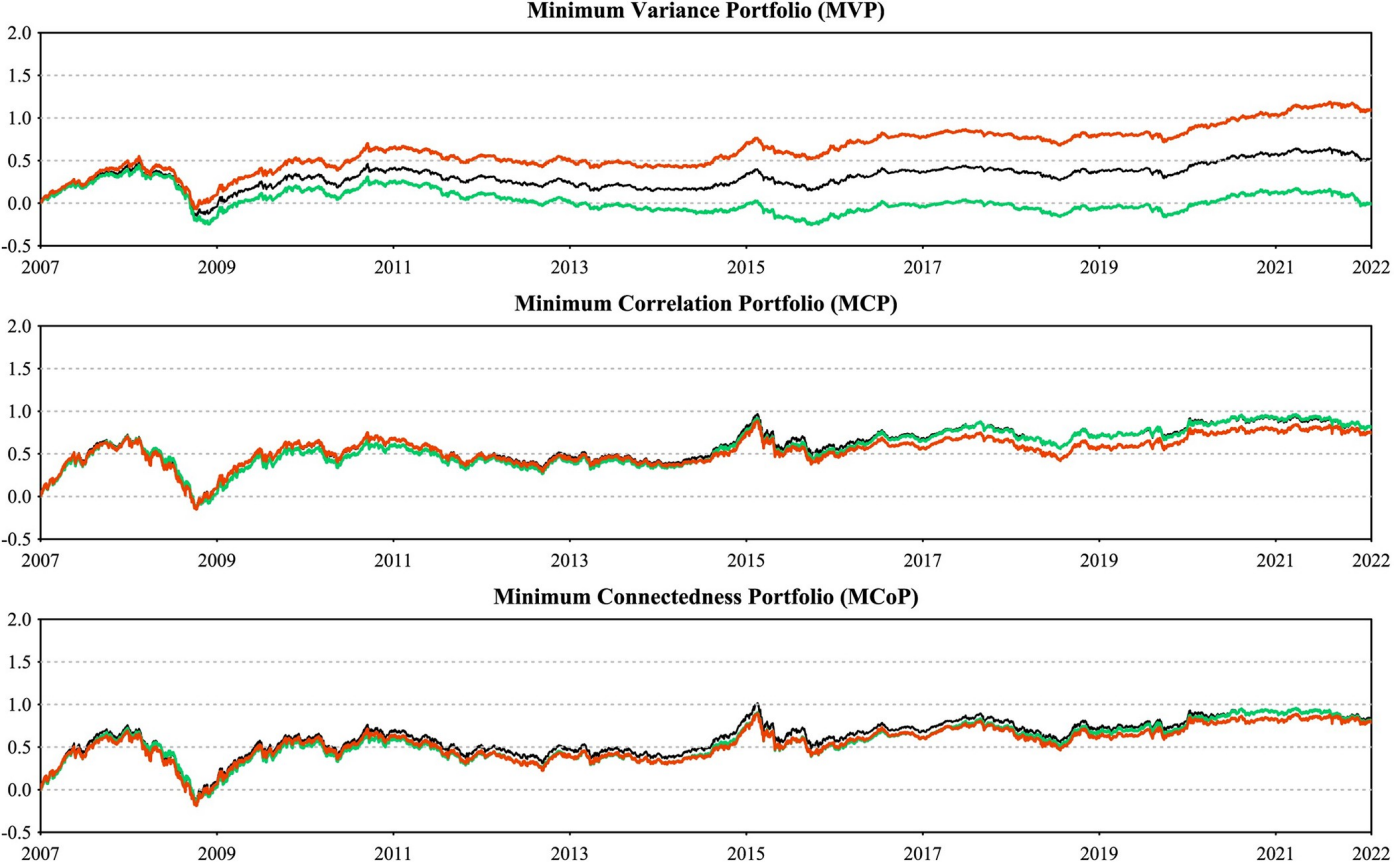
Asymmetric cumulative returns on portfolios. Results are based on the time-varying variance-covariance matrices of the TVP-VAR model with a lag length of order one (BIC) and a generalized variance decomposition of 20-step-ahead forecast error.

**Fig 13:**
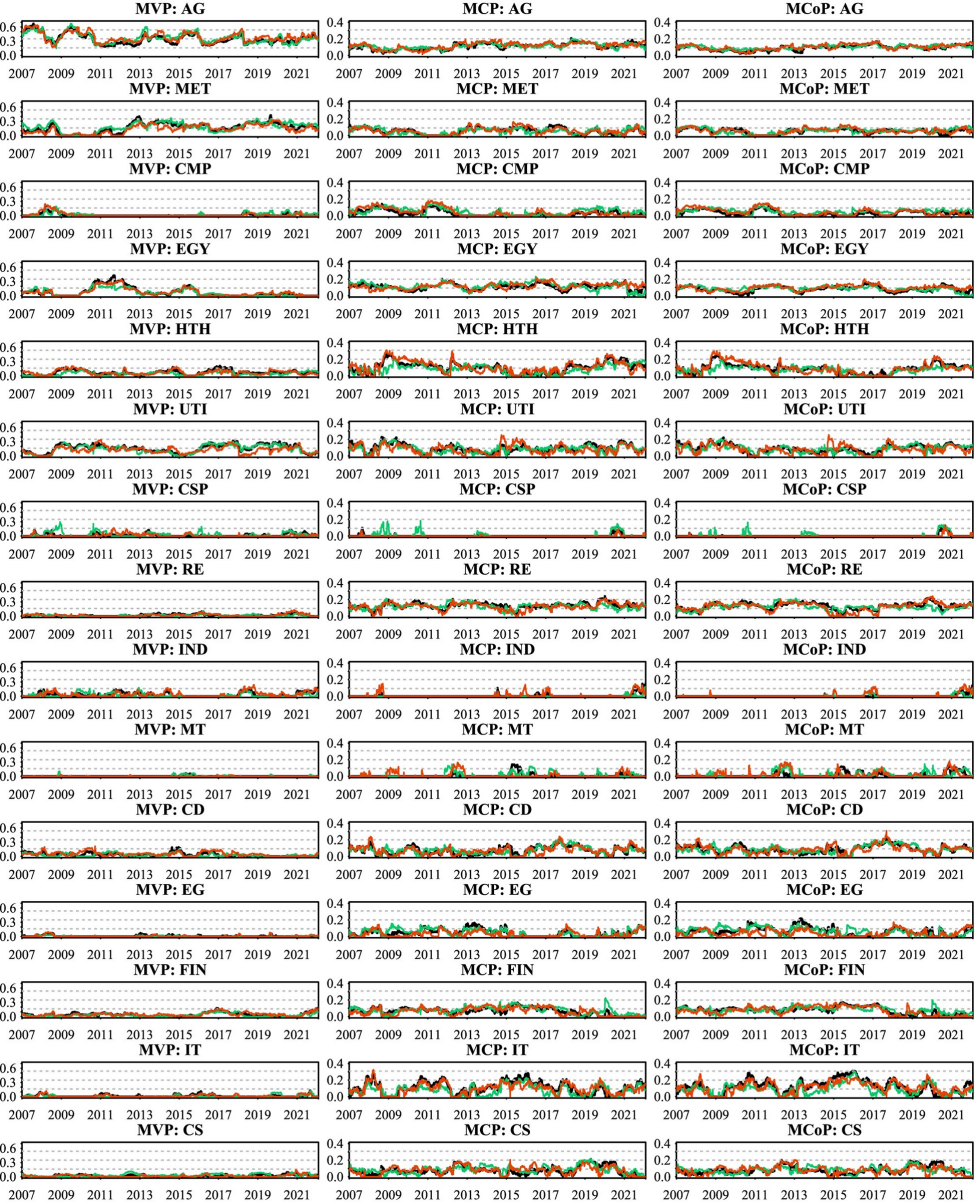
Dynamic multivariate portfolio weights. Results are based on the time-varying variance-covariance matrices of the TVP-VAR model with a lag length of order one (BIC) and a generalized variance decomposition of 20-step-ahead forecast error.

Fig 12 illustrates the asymmetric cumulative returns of portfolios constructed using the three methods. The first graph depicts the cumulative return performance of the minimum variance portfolio (MVP). The second graph illustrates the cumulative return performance of the minimum correlation portfolio (MCP), while the third graph presents the cumulative return performance of the minimum connectedness Portfolio (MCoP). While these portfolios exhibit a consistent growth trend, they face pronounced setbacks during turbulent periods, such as those in 2008, 2015, and 2020. Notably, our analysis reveals pronounced asymmetry in cumulative returns during periods of positive and negative market performance. Negative returns contribute more significantly to the MVP, underscoring the importance of considering return asymmetry in portfolio construction. To provide a deeper understanding of each portfolio’s composition, we present the dynamic weights of these portfolios in Fig 13. These dynamic weights illustrate how the portfolios adjust over time based on changing market conditions. Notably, they reflect the distinctions in positive and negative return-based signals, emphasizing the inherent asymmetry of return spillovers.

We further examine the efficiency of hedging for the three portfolios. Table 5 shows the ability to create an MVP through a specific asset allocation strategy. In the context of prevailing market conditions, it is evident that an investment distribution characterized by an average allocation of 35% in AG, 17% in MET, 1% in CMP, 7% in EGY, 7% in HTH, 15% in UTI, 2% in CSP, 2% in RE, 3% in IND, 3% in CD, and 1% in EG, along with 4% in FIN, 1% in IT, and 2% in CS, would significantly reduce the volatility of each asset in this portfolio by 11%, 53%, 66%, 72%, 81%, 77%, 81%, 86%, 81%, 83%, 80%, 84%, 82%, and 86%. These reductions in volatility are both financially and statistically significant, reaching statistical significance at the 0.1% level. The results remain consistent when examining portfolios based on positive and negative returns, indicating robustness in average weights and hedging efficiency patterns. However, these findings do not diminish the significance of asymmetry but provide additional information on this matter. While risk-averse investors can assess the inter-asset equilibrium to determine which investments minimize risk, and risk-tolerant investors can remain indifferent to the same [17, 67], there are no asymmetric dynamics in measuring and comparing positive and negative returns that investors choose to consider when making investment decisions.

**Table 5:** Minimum variance portfolio weights.

	Mean	Std.Dev.	5%	95%	HE	p-value
AG	0.35	0.11	0.21	0.56	0.11	0.00
MET	0.17	0.10	0.00	0.30	0.53	0.00
CMP	0.01	0.03	0.00	0.08	0.66	0.00
EGY	0.07	0.10	0.00	0.31	0.72	0.00
HTH	0.07	0.05	0.00	0.16	0.81	0.00
UTI	0.15	0.08	0.00	0.27	0.77	0.00
CSP	0.02	0.03	0.00	0.08	0.81	0.00
RE	0.02	0.02	0.00	0.06	0.86	0.00
IND	0.03	0.04	0.00	0.12	0.81	0.00
MT	0.00	0.01	0.00	0.00	0.83	0.00
CD	0.03	0.04	0.00	0.10	0.80	0.00
EG	0.01	0.01	0.00	0.04	0.84	0.00
FIN	0.04	0.04	0.00	0.12	0.82	0.00
IT	0.01	0.02	0.00	0.06	0.86	0.00
CS	0.02	0.02	0.00	0.06	0.86	0.00
+	Mean	Std.Dev.	5%	95%	HE	p-value
AG	0.37	0.11	0.23	0.58	0.10	0.00
MET	0.18	0.09	0.00	0.31	0.52	0.00
CMP	0.02	0.03	0.00	0.09	0.65	0.00
EGY	0.06	0.07	0.00	0.19	0.71	0.00
HTH	0.05	0.03	0.00	0.10	0.81	0.00
UTI	0.14	0.08	0.00	0.26	0.76	0.00
CSP	0.03	0.05	0.00	0.12	0.81	0.00
RE	0.01	0.02	0.00	0.04	0.86	0.00
IND	0.02	0.04	0.00	0.11	0.81	0.00
MT	0.00	0.01	0.00	0.04	0.83	0.00
CD	0.03	0.03	0.00	0.09	0.80	0.00
EG	0.00	0.01	0.00	0.02	0.84	0.00
FIN	0.03	0.03	0.00	0.10	0.82	0.00
IT	0.01	0.02	0.00	0.06	0.86	0.00
CS	0.03	0.03	0.00	0.08	0.85	0.00
-	Mean	Std.Dev.	5%	95%	HE	p-value
AG	0.38	0.10	0.24	0.57	0.05	0.15
MET	0.14	0.09	0.00	0.27	0.50	0.00
CMP	0.02	0.04	0.00	0.11	0.63	0.00
EGY	0.07	0.08	0.00	0.27	0.70	0.00
HTH	0.07	0.05	0.00	0.16	0.80	0.00
UTI	0.12	0.07	0.01	0.26	0.75	0.00
CSP	0.01	0.03	0.00	0.07	0.79	0.00
RE	0.02	0.03	0.00	0.07	0.85	0.00
IND	0.04	0.05	0.00	0.14	0.80	0.00
MT	0.00	0.00	0.00	0.00	0.82	0.00
CD	0.05	0.04	0.00	0.13	0.78	0.00
EG	0.01	0.02	0.00	0.04	0.83	0.00
FIN	0.05	0.04	0.00	0.13	0.81	0.00
IT	0.01	0.02	0.00	0.07	0.85	0.00
CS	0.02	0.02	0.00	0.06	0.84	0.00

Results are based on Markovitz [65].

Table 6 presents the average weight allocation and hedging efficiency of the MCP. These results substantially differ from those of the MVP. Notably, an average allocation of 11% in AG, 5% in MET, 3% in CMP, 11% in EGY, 10% in HTH, 9% in UTI, 0 in CSP, 13% in RE, 1% in IND, 1% in CD, 7% in EG, 4% in FIN, 7% in IT, and 8% in CS, would result in a statistically significant reduction in the volatility of each asset in this portfolio by -122%, -17%, 15%, 29%, 53%, 42%, 52%, 65%, 53%, 59%, 50%, 61%, 55%, 65%, 64%. These results indicate that the MCP methodology, which focuses on minimizing inter-asset correlation, constructs portfolios in which each asset contributes less to reducing portfolio volatility than the MVP methodology [67]. Furthermore, it is also worth noting that the calculated negative hedge efficiency values for AG and MET imply that including these commodities in a portfolio can increase both the volatility and the risk associated with each asset in the portfolio. This observation underscores the importance of careful asset selection and allocation, especially considering commodities such as AG and MET.

**Table 6:** Minimum variance portfolio weights.

	Mean	Std.Dev.	5%	95%	HE	p-value
AG	0.11	0.03	0.05	0.16	-1.22	0.00
MET	0.05	0.04	0.00	0.11	-0.17	0.00
CMP	0.03	0.03	0.00	0.10	0.15	0.00
EGY	0.11	0.03	0.05	0.17	0.29	0.00
HTH	0.10	0.06	0.00	0.21	0.53	0.00
UTI	0.09	0.05	0.02	0.17	0.42	0.00
CSP	0.00	0.01	0.00	0.03	0.52	0.00
RE	0.13	0.04	0.06	0.18	0.65	0.00
IND	0.01	0.02	0.00	0.04	0.53	0.00
MT	0.01	0.02	0.00	0.05	0.59	0.00
CD	0.07	0.05	0.00	0.15	0.50	0.00
EG	0.04	0.04	0.00	0.12	0.61	0.00
FIN	0.07	0.04	0.00	0.13	0.55	0.00
IT	0.11	0.07	0.00	0.23	0.65	0.00
CS	0.08	0.05	0.00	0.18	0.64	0.00
+	Mean	Std.Dev.	5%	95%	HE	p-value
AG	0.11	0.03	0.06	0.16	-1.11	0.00
MET	0.05	0.03	0.00	0.10	-0.12	0.00
CMP	0.05	0.04	0.00	0.12	0.19	0.00
EGY	0.11	0.04	0.03	0.18	0.33	0.00
HTH	0.09	0.05	0.00	0.17	0.56	0.00
UTI	0.09	0.04	0.02	0.16	0.45	0.00
CSP	0.01	0.03	0.00	0.09	0.54	0.00
RE	0.12	0.03	0.06	0.17	0.67	0.00
IND	0.00	0.01	0.00	0.00	0.55	0.00
MT	0.01	0.03	0.00	0.08	0.61	0.00
CD	0.07	0.04	0.00	0.15	0.52	0.00
EG	0.05	0.04	0.00	0.11	0.62	0.00
FIN	0.08	0.04	0.00	0.14	0.57	0.00
IT	0.08	0.06	0.00	0.18	0.66	0.00
CS	0.09	0.04	0.03	0.17	0.66	0.00
-	Mean	Std.Dev.	5%	95%	HE	p-value
AG	0.12	0.04	0.05	0.17	-1.14	0.00
MET	0.05	0.04	0.00	0.13	-0.13	0.00
CMP	0.04	0.05	0.00	0.15	0.18	0.00
EGY	0.12	0.03	0.06	0.17	0.32	0.00
HTH	0.11	0.07	0.00	0.24	0.55	0.00
UTI	0.08	0.05	0.00	0.17	0.44	0.00
CSP	0.00	0.01	0.00	0.00	0.54	0.00
RE	0.12	0.04	0.04	0.17	0.66	0.00
IND	0.01	0.03	0.00	0.08	0.54	0.00
MT	0.01	0.03	0.00	0.09	0.60	0.00
CD	0.07	0.05	0.00	0.15	0.52	0.00
EG	0.03	0.04	0.00	0.10	0.62	0.00
FIN	0.06	0.05	0.00	0.13	0.56	0.00
IT	0.10	0.06	0.00	0.19	0.66	0.00
CS	0.08	0.05	0.00	0.16	0.65	0.00

Results are based on Christoffersen et al. [66].

Turning to the average weight allocation and hedging efficiency of the minimum connectedness portfolio in Table 7. Remarkably, this portfolio closely resembles the MCP presented in Table 6. This similarity is likely due to their shared time-varying variance-covariance matrix. However, a detailed analysis of the portfolio’s performance under different scenarios reveals notable differences.

**Table 7:** Minimum variance portfolio weights.

	Mean	Std.Dev.	5%	95%	HE	p-value
AG	0.09	0.03	0.04	0.13	-1.39	0.00
MET	0.05	0.03	0.00	0.09	-0.26	0.00
CMP	0.03	0.03	0.00	0.09	0.08	0.01
EGY	0.08	0.03	0.03	0.13	0.24	0.00
HTH	0.09	0.05	0.00	0.19	0.50	0.00
UTI	0.09	0.04	0.02	0.16	0.37	0.00
CSP	0.00	0.02	0.00	0.01	0.48	0.00
RE	0.12	0.04	0.06	0.18	0.62	0.00
IND	0.00	0.01	0.00	0.03	0.49	0.00
MT	0.01	0.03	0.00	0.08	0.55	0.00
CD	0.09	0.04	0.02	0.16	0.46	0.00
EG	0.06	0.05	0.00	0.15	0.57	0.00
FIN	0.07	0.04	0.00	0.14	0.51	0.00
IT	0.12	0.08	0.00	0.26	0.62	0.00
CS	0.08	0.04	0.01	0.17	0.61	0.00
+	Mean	Std.Dev.	5%	95%	HE	p-value
AG	0.10	0.02	0.06	0.14	-1.23	0.00
MET	0.06	0.03	0.00	0.11	-0.18	0.00
CMP	0.05	0.03	0.01	0.10	0.14	0.00
EGY	0.08	0.03	0.03	0.12	0.29	0.00
HTH	0.07	0.04	0.00	0.13	0.53	0.00
UTI	0.08	0.04	0.01	0.15	0.42	0.00
CSP	0.01	0.03	0.00	0.07	0.52	0.00
RE	0.11	0.04	0.05	0.18	0.65	0.00
IND	0.00	0.01	0.00	0.04	0.52	0.00
MT	0.02	0.03	0.00	0.10	0.58	0.00
CD	0.09	0.04	0.02	0.17	0.50	0.00
EG	0.06	0.04	0.00	0.13	0.60	0.00
FIN	0.08	0.03	0.02	0.14	0.55	0.00
IT	0.09	0.06	0.00	0.19	0.65	0.00
CS	0.08	0.04	0.02	0.15	0.64	0.00
-	Mean	Std.Dev.	5%	95%	HE	p-value
AG	0.10	0.03	0.05	0.15	-1.26	0.00
MET	0.06	0.03	0.00	0.11	-0.19	0.00
CMP	0.04	0.04	0.00	0.13	0.13	0.00
EGY	0.09	0.03	0.04	0.14	0.28	0.00
HTH	0.09	0.07	0.00	0.22	0.52	0.00
UTI	0.08	0.05	0.00	0.17	0.41	0.00
CSP	0.00	0.01	0.00	0.00	0.51	0.00
RE	0.11	0.05	0.01	0.17	0.64	0.00
IND	0.01	0.02	0.00	0.07	0.52	0.00
MT	0.02	0.04	0.00	0.12	0.58	0.00
CD	0.09	0.05	0.01	0.17	0.49	0.00
EG	0.04	0.04	0.00	0.12	0.60	0.00
FIN	0.07	0.05	0.00	0.15	0.54	0.00
IT	0.11	0.07	0.00	0.22	0.64	0.00
CS	0.08	0.05	0.00	0.16	0.63	0.00

Results are based on Broadstock et al. [67].

Finally, Table 8 presents the average returns, standard deviations and sharpe ratios of portfolios constructed using different techniques for overall returns, positive returns, and negative returns. These results are reported for the entire sample period and sub-periods, such as the pre-2008 global financial crisis (pre-GFC), the 2008 global financial crisis (GFC), the subsequent recovery period, the 2015 Chinese stock market crisis (CSMC), the COVID-19 pandemic, the post-pandemic era, and the recent Russia-Ukraine war (R-U war). Our results highlight the superiority of the MCoP. It achieves the highest average return (0.029%) over the sample period, followed by the MVP (0.013%) and the MCP (-0.003%). The MCoP excels in managing asymmetric returns during economic downturns (GFC, CSMC, COVID-19 pandemic, and R-U war), effectively minimizing losses. During economic upswings (pre-GFC, recovery, post-pandemic), the MCoP maintains robust average returns and Sharpe ratios, demonstrating its ability to capture the impact of asymmetric returns on portfolio design. In conclusion, the MCoP emerges as a top-performing approach, consistent with previous research (Adekoya et al. [17], Broadstock et al. [67], and Abdullah et al. [10]). These results provide valuable insights for investors and portfolio managers navigating complex market dynamics and optimizing risk-return profiles.

**Table 8:** Portfolio performance.

	Whole period			pre-GFC			GFC			Recovery period		
	MVP	MCP	MCop	MVP	MCP	MCop	MVP	MCP	MCop	MVP	MCP	MCop
Mean	0.013	-0.0003	0.029	0.178	0.159	0.200	-0.293	-0.297	-0.276	0.099	0.087	0.126
Std.Dev.	0.815	0.821	0.841	0.813	0.800	0.839	1.464	1.410	1.544	1.101	1.099	1.115
SR	0.016	-0.0004	0.034	0.220	0.199	0.238	-0.200	-0.211	-0.179	0.090	0.079	0.113
+												
Mean	0.021	0.021	0.019	0.290	0.285	0.282	-0.423	-0.418	-0.420	0.144	0.134	0.148
Std.Dev.	1.289	1.253	1.267	1.530	1.443	1.478	2.205	2.086	2.263	1.555	1.519	1.529
SR	0.016	0.017	0.015	0.190	0.198	0.191	-0.192	-0.201	-0.186	0.092	0.088	0.097
-												
Mean	0.022	0.021	0.021	0.303	0.289	0.276	-0.440	-0.431	-0.433	0.146	0.134	0.145
Std.Dev.	1.339	1.293	1.301	1.566	1.517	1.453	2.257	2.095	2.293	1.616	1.570	1.564
SR	0.016	0.016	0.016	0.194	0.190	0.190	-0.195	-0.206	-0.189	0.090	0.085	0.092
	CSMC			COVID-19 pandemic			post-pandemic			R-U war		
	MVP	MCP	MCop	MVP	MCP	MCop	MVP	MCP	MCop	MVP	MCP	MCop
Mean	-0.178	-0.242	-0.171	-0.161	-0.227	-0.156	0.117	0.129	0.122	-0.096	-0.122	-0.059
Std.Dev.	0.938	0.987	0.972	1.295	1.360	1.295	0.661	0.683	0.681	0.934	0.991	0.922
SR	-0.189	-0.245	-0.176	-0.124	-0.167	-0.121	0.177	0.189	0.180	-0.103	-0.124	-0.064
+												
Mean	-0.406	-0.412	-0.389	-0.114	-0.202	-0.104	0.117	0.135	0.115	-0.076	-0.087	-0.059
Std.Dev.	2.487	2.367	2.373	1.868	1.928	1.802	0.973	0.969	0.958	1.188	1.170	1.188
SR	-0.163	-0.174	-0.164	-0.061	-0.105	-0.058	0.120	0.139	0.120	-0.064	-0.074	-0.049
-												
Mean	-0.449	-0.421	-0.386	-0.130	-0.186	-0.115	0.122	0.146	0.115	-0.078	-0.090	-0.054
Std.Dev.	2.553	2.463	2.434	1.962	2.025	1.892	1.009	0.987	0.985	1.200	1.197	1.201
SR	-0.176	-0.171	-0.159	-0.066	-0.092	-0.061	0.120	0.147	0.116	-0.065	-0.075	-0.045

MVP, MCP, MCoP, and SR stand for the minimum variance portfolio [65], minimum correlation portfolio [66], minimum connectedness portfolio [67], and Sharpe ratio [68], respectively.

## 6 Conclusion

This study examines the asymmetric return spillovers and connectedness between sectoral commodities and industry stocks in China’s financial market. The results show significant time-varying spillovers between sectoral commodities and industry stocks, with the commodity market acting as a net recipient of return spillovers from the stock market, indicating that the stock market plays a crucial role in transmitting information to the commodity market and influencing commodity prices. This dynamic connectedness was not homogenous across all sectors, with the materials (MT), energy (EG), and industrials (IND) stock sectors being the most significant contributors to return spillovers for the four sectoral commodities. This likely results from the integral role these sectors play in producing or processing commodities, suggesting that production linkages or demand intensity play a role in establishing the connectedness between commodities and stocks. Moreover, the financials (FIN) and consumer discretionary (CSP) stock sectors also emerged as notable contributors, ranking fourth and fifth, respectively, as sources of net return spillovers to all sectoral commodities except agricultural (AGR) futures. This highlights these spillovers’ pervasive nature and sectoral heterogeneity, which extend beyond the immediate production relationship into the broader financial sector and consumer markets. Furthermore, we captured significant asymmetric connectedness characterized by the dominance of negative return spillovers, influenced by major economic and political events such as the 2008 financial crisis, the 2015 stock market crisis, the COVID-19 pandemic, and the Russia-Ukraine war. Unexpectedly, negative return spillovers did not dominate during periods of financial turbulence. Instead, events such as the COVID-19 pandemic led to an increase in net directional connectedness and a shift towards negative return dominance. This highlights the complex dynamics of connectedness and the influence of specific events on asymmetric market connectedness. Finally, the portfolio analysis shows that the minimum connectedness portfolio outperforms other approaches and better captures the differences in return asymmetries. Beyond interconnectedness, the portfolio analysis results clearly favored the minimum connectedness portfolio method, consistently demonstrating superior performance across different scenarios and emphasizing its effectiveness in capturing the influence of asymmetric returns on portfolio design.

By comparing our findings to previous studies, we contribute to the literature by focusing on the specific dynamics and asymmetries within the Chinese market. While some studies have studied the interconnectedness between commodities and stock markets in other contexts, our sector-level analysis reduces the influence of specific factors affecting individual commodities. It reveals potential sectoral heterogeneity that may be masked. Moreover, we have revealed that the relationship between commodities and stock markets in China’s commodity financialization is primarily driven by net information spillovers from the stock market to the commodity market, in contrast to the notion of strong bidirectional commodity financialization observed in other markets. This highlights the unique characteristics of China’s financial market, characterized by a lack of significant commodity investment vehicles and the prevalence of retail investors in the futures market, leading to a distinct absence of true commodity financialization.

In conclusion, the primary objective of this study was to explore the intricate relationships between sectoral commodities and industry stocks in China’s financial market and to analyze the directional connectedness and asymmetric return spillovers between these sectors. The results confirm the hypothesis of a deep connectedness between sectoral commodities and industry stocks and shed light on the asymmetric nature of these spillovers and their temporal variation. First, policymakers should recognize the event-driven nature of the dynamic and asymmetric connectedness between China’s commodity market and the stock markets. Understanding these dynamics can help develop effective risk management strategies and policies to mitigate significant events’ impact on the market. Second, the significant difference in the connectedness between sectoral commodities and industry stocks suggests that policies and trading strategies should be sector-specific. A one-size-fits-all approach may not be the most effective when dealing with the excessive risk spillovers across China’s commodity and stock markets. Instead, adopting an approach that takes into account the unique characteristics of different sectors may lead to more effective strategies. Finally, for investors, recognizing the unique characteristics of China’s commodity and stock markets can provide valuable insights for portfolio diversification strategies, especially during periods of economic uncertainty.

Future studies could delve deeper into the underlying mechanisms driving the spillovers and examine the role of market microstructure factors, macroeconomic factors, and investor behavior in the interconnectedness.

## Supporting information

S1 Data (XLSX)
